# Introducing FREM_ML_: a decision-support approach for automated identification of individuals at high imminent fracture risk

**DOI:** 10.1007/s11657-025-01613-5

**Published:** 2025-11-05

**Authors:** Marlene Rietz, Jan C. Brønd, Sören Möller, Jens Søndergaard, Bo Abrahamsen, Katrine Hass Rubin

**Affiliations:** 1 Research Unit OPEN, Department of Clinical Research, University of Southern Denmark, Odense, Denmark; 2 Present Address: Steno Diabetes Center Odense, Department of Clinical Research, University of Southern Denmark, Odense, Denmark; 3 Division of Clinical Physiology, Department of Laboratory Medicine, Karolinska Institutet, Huddinge, Sweden; 4 OPEN - Open Patient Data Explorative Network, Odense University Hospital, Odense, Denmark; 5 Research Unit for Epidemiology, Biostatistics and Biodemography, Department of Public Health, University of Southern Denmark, Odense, Denmark; 6 Research Unit for General Practice, Department of Public Health, University of Southern Denmark, Odense-Esbjerg, Denmark

**Keywords:** Osteoporosis, Major osteoporotic fractures, Machine learning, Decision-support, Case-finding, Explainable AI

## Abstract

**Summary:**

This study used explainable AI to improve the Danish FREM model for predicting one-year risk of major osteoporotic fractures in over 2.4 million individuals aged ≥ 45. A DART boosting algorithm improved performance (AUC 0.77), with explainable outputs aiding clinical interpretation and guiding referrals for fracture risk assessment.

**Purpose:**

This study aimed to use explainable artificial intelligence to improve the Fracture Risk Evaluation Model (FREM), in the prediction of imminent (one-year) risk of major osteoporotic fractures (MOFs).

**Methods:**

FREM_ML_ was trained and validated using complete registry data extracted for the Danish population ≥ 45 years without previous osteoporosis diagnoses or treatment (N = 2,438,140). A Dropouts meet multiple Additive Regression Tree (DART) boosting algorithm was used. Predictors of MOFs (2022), automatically extracted for the 15-year lookback period (2007–2021), included hospital diagnoses, filled medication prescriptions, days since the last redemption of medications specific to fall and osteoporosis risk, as well as markers of polypharmacy and multi-morbidity. Stratified analyses were carried out, and model outputs were evaluated in the context of explainable artificial intelligence (AI).

**Results:**

FREM_ML_ displayed an overall area under the curve (95% confidence interval) of 0.77 (0.76, 0.77) – making it superior to previous versions of FREM. While age and sex were the most relevant predictors of MOF events, advanced feature engineering, including temporal information, contributed to model performance. Importantly, stratified analyses highlighted changing model performance across age groups and poorer prediction performance in males. Shapley Additive exPlanations values, a feature importance metric in explainable AI, facilitated clinical interpretation of relative MOF risk.

**Conclusion:**

The publicly available FREM_ML_ boosting model, combined with explainable AI, may be an effective decision support approach in a physician’s referral of individuals at high imminent risk of fractures to dual-energy X-ray absorptiometry.

**Supplementary Information:**

The online version contains supplementary material available at 10.1007/s11657-025-01613-5.

## Introduction

Major osteoporotic fractures (MOFs) are severe health complications associated with a radical loss of health-related quality of life [1] and a high risk of mortality [2]. Several medical diagnoses, clinical risk factors, and medications may increase the risk of serious falls [3, 4] and/or low bone mineral density (BMD) [5]. Osteoporosis, a major driver of any type of fracture, requires a dual x-ray absorptiometry (DXA) for diagnosis [6] and – depending on national guidelines – treatment [7]. Therefore, algorithms to predict the risk of MOF and target DXA imaging may protect quality of life in people with low BMD while also reducing medical expenses.

A variety of risk prediction methodologies have been introduced to estimate the 1- to 10-year risk of fractures; with FRAX® most commonly employed to estimate the 10-year risk of MOFs [8]. Other tools include QFracture and CFracture [9] and the Garvan Fracture Risk Calculator [10], and most tools require manual data entry describing anamneses and/or clinical assessments. To automatically estimate imminent (one-year) fracture risk and propose interventions, we have previously developed the Fracture Risk Evaluation Model (FREM) [11]. FREM_ver1_ was entirely based on registered hospital diagnoses throughout a 15-year look-back period obtained directly from electronic health records (eHRs). The risk prediction has been validated in Denmark [12] and Canada [13] and was recently refined by adding data describing medication exposure [14]. Next to FREM, the Crystal Bone algorithm (Amgen®) is also exclusively based on electronic health records and employs natural-language processing-based machine learning (ML) methods [15]. A more traditional statistical model based on registry data, exclusively, has recently been suggested by Swedish researchers for the isolated prediction of the five-year risk of hip fractures [16].

In contrast to standard regression-based models, gradient boosting allows for non-linear modelling. For instance, a gradient boosting model (GBM) has recently demonstrated promising results for the prediction of cerebral infarctions [17]. Indeed, register-based data is highly complex and non-linear modelling may be leveraged for accurate personalized predictions of imminent MOFs. Combined with explainable artificial intelligence (XAI) methodology, this may facilitate risk-guided DXA diagnostics in in primary care within a decision-support tool [18].

## Purpose

This study aimed to develop and validate a gradient boosting machine learning model to predict the imminent (one-year) risk of MOFs in the Danish population ≥ 45 years using data describing previous diagnoses and filled prescriptions from national health registers and to explore potential implementation strategies.

## Methods

### Data sources

The FREM algorithms are dependent on registry data extracted from the Civil Registration System (CRS)[19], the Danish National Patient Register (NPR) [20] and the Danish National Prescription Register (DNPR) [21]. In Denmark, data extracted from national registers is linked using unique personal identification numbers, ensuring high-quality data linkage [19]. For this study, diagnoses encoded in accordance with the International Classification of Diseases (ICD) in the NPR as well as anatomical therapeutic chemical (ATC) codes and redemption dates for filled prescriptions in the DNPR were obtained. Next, this information was linked to unique identification numbers, age, registered gender, employed as a proxy of biological sex, and death and emigration data obtained from the CRS. Over the counter (OTC) medications were not captured.

### Study population and model cohorts

The FREM population was compiled from Danish register data collected from 01 January 2007–31 December 2022 and included all individuals registered in Denmark ≥ 45 years at the index date, 01 January 2022. Individuals with either a diagnosis or treatment of osteoporosis during the 15-year look-back period (Supplementary Table S1), as well as those with an incomplete look-back period, were excluded [22].

### Model components

- Outcomes (fractures)

The primary outcome is MOF, defined as a fracture of the humerus, the clinical vertebral area, the forearm, or hip followed by a relevant imaging procedure and/or surgery within seven days of hospitalization [23]. For individuals with several fractures reported in 2022, the first fracture was considered. The risk of a hip fracture, an incident fracture (ICD10: S720, S721 or S722) followed by a relevant surgical procedure (NFB* or NFJ4-9) within seven days of hospitalization, was explored as a secondary outcome [23].

- Predictors of fractures

All determinants of fractures were extracted from data collected during the 15-year look-back period between January 01, 2007, and December 31, 2021. Initially, all diagnoses as well as prescriptions were considered as potential features.

Several diagnosis feature types were compiled using feature engineering (Supplementary Table S2). Initially, all diagnosis codes were used to create binary features describing whether an individual was diagnosed with a specific ICD-10 diagnosis category. Furthermore, a variety of diagnosis-based risk factors for osteoporotic fractures were compiled (Supplementary Table S3). The number of individuals assigned to each risk factors is presented in Supplementary Table S4. Next, multi-morbidity significantly contributes to the risk of fractures [24]. Therefore, three features describing the number of diagnoses within unique ICD-10 alpha codes were included. A total of three exposure windows corresponding to diagnoses within one year before the index date, 2–5 years before the index date, and 6–15 years before the index date were used, in accordance with Bruun-Jensen et al.[17] Furthermore, the Charlson Comorbidity Index (CCI) [25] was calculated for each individual.

Medications were divided a priori into pharmacological agents increasing the risk of osteoporosis, pharmacological agents increasing the risk of fracture-inducing falls, and pharmacological agents likely functioning as proxies for missing or incomplete diagnosis codes (Supplementary Table S5) [22].

Diagnosis proxies were generated as a binary variable indicating use of a 2nd level ATC therapeutic subgroup. Osteoporosis- and fall-risk medications were compiled using days from last redemption of a 4th level ATC chemical subgroup. All individuals without a registered redemption were assigned the complete duration (15 years) of the look-back period. In Supplementary Table S6, the number of individuals using included medications are presented.

As association of fall-risk medication and fractures may be strongest upon recent exposure, additional binary features describing a registered redemption within six months from the index were created.

Finally, a polypharmacy proxy was introduced to address an association between polypharmacy and fracture risk, computing the total count of unique 2nd level ATC therapeutic subgroups redeemed during the look-back period [4].

### Model development

All model development, validation, and statistical analyses was carried out in R (Version 4.3.3) via R.Studio (Version 2024.4.2.764). The sample was divided into a 60% training cohort, a 20% validation cohort, and a 20% testing cohort stratified by age and sex. No differences were reported between the training, validation, and testing cohort (Supplementary Table S7). To remove variables with collinearity and zero variance within the cohorts, functions from the *caret* [26] R package were used.

### Model selection

The primary model examined in this study uses the Dropouts meet Multiple Additive Regression Tree (DART) boosting algorithm [27]. A hyperparameter search was carried out in the training data to optimize hyperparameters for each model (Supplementary Table S8). Following this, ten-fold cross-validation with a maximum of 2000 training rounds was employed to evaluate model performance and perform final tuning. Lastly, the final model was trained on both the training and validation cohort.

### Model evaluation

Model performance was evaluated in the testing cohort using area under the curve (AUC) and 95% confidence intervals (CI) as a primary metric. A Youden’s index maximising the sum of sensitivity and specificity, computed in the training and validation cohort, was used to classify individuals with a high risk of fracture in the testing sample. Model analytics for the Youden’s threshold and other cut-offs were computed, Supplementary Table S9. Density plots were graphed across fracture labels, showing the distribution of probabilities across cases. The final model stucture was made available in a public repository (Link https://osf.io/hz58k/?view_only=364f578ccb49413384be253216687680).

Mean Shapley Additive exPlanations (SHAP) values were calculated for all model features. Only SHAP values and sample means of the 20 most relevant features were visualized*.* A negative SHAP value indicates that a feature lowers fracture risk. If low feature values are associated with negative SHAP values, it suggests that lower feature values tend to decrease fracture risk. The absolute value of a SHAP value, i.e., its distance from zero, provides insights on the size of the impact on risk estimates. A detailed description discussing the XAI methodology using SHAP values can be found in Supplementary Method Section SM1.

In subgroup analyses, the performance of the FREM_ML_ model across age and sex, dominating predictors of fracture risk in the original FREM risk prediction model, was assessed [11]. Two sex-stratified models were evaluated. Additionally, age- and sex-specific risk cutoffs for the non-stratified model were computed, corresponding relative fracture risks were estimated, and the sensitivity and specificity of these cutoffs were reported.

## Results

A total of 2,438,140 individuals qualified for inclusion. Briefly, 55,040 individuals displayed an incomplete look-back period and 247,105 (79.3% female) individuals were excluded due to a previous osteoporosis diagnosis and/or medication.

During the index year (2022), 15,031 (1.3%) MOF were identified among 1,198,216 females, and 7,011 (0.6%) among 1,239,924 males. Of those, 3,478 (23.1%) and 2,400 (34.2%) cases in females and males corresponded to HFs, respectively. Furthermore, 42,614 (3.6%) females and 15,688 (1.3%) males underwent a DXA assessment in 2022, and 18,045 (1.5%) females and 6,333 (0.5%) males received a primary osteoporosis diagnosis or first instance of an anti-osteoporotic medication (AOM).

The cleaned dataset included 1,900 features. For the stratified models, 1,862 and 1,794 features were included for female and male individuals, respectively.

Baseline characteristics recorded during the look-back period describing the thirty most relevant features included in the non-stratified model are presented in Table 1. Briefly, female and male individuals who experienced a MOF in 2022 had a median age (interquartile range [IQR]) of 72.8 (18.5) and 72.0 (20.1), respectively, resulting in an approximate median age difference of 10 years, compared to individuals without a MOF event. Hospital diagnoses most relevant for predicted MOF risk were several fractures, dislocations, coxathrosis, as well as an open wound of the head – suggesting previous falls, Table 1. The alcohol-related risk factor was more than three times as common in individuals with registered MOFs, corresponding to 10.1% in male and 3.2% in female individuals who experienced a MOF in 2022. Furthermore, individuals with MOFs were more likely to take drugs for constipation and anemia, and males who experienced a MOF were 4.6% more likely to have received systemic antibacterials in the look-back period. Days since the last redemption of medications specific to fall and osteoporosis risk are presented across sex and exposure windows in Table 1. Indeed, proton pump inhibitors, classified as medication posing a risk of osteoporosis, were redeemed less than one year from the index date by 8.9% more male and 6.6% more female individuals in the MOF sample compared to individuals who did not experience a fracture in the index year. Considering fall risk, thiazide and potassium combinations, sulfonamides, angiotensin 2-receptor blockers, selective serotonin reupdake inhibitiors, and other antidepressants were important components of the FREM_ML_ model.

**Table 1:** Descriptive information on relevant model features at the index date (01.01.2022) and throughout the look-back period across sex and MOF diagnoses during the index year

	**Follow-up status**			**No MOF**		**MOF**	
	Sample size			1,232,913	1,183,185	7011	15,031
	Sex			Male	Female	Male	Female
	Age (years)*			61.5 (18.3)	61.2 (18.7)	72.0 (20.1)	72.8 (18.5)
	Charlson Comorbidity Index*			0.0 (0.0)	0.0 (0.0)	0.0 (2.0)	0.0 (1.0)
	Deaths			23,819 (1.9)	17,154 (1.4)	814 (11.6)	884 (5.9)
	Emigrations			2192 (0.2)	1204 (0.1)	3 (0.0)	3 (0.0)
	Hip fractures			0 (0.0)	0 (0.0)	2400 (34.2)	3478 (23.1)
	Coxarthrosis		M16	50,510 (4.1)	53,911 (4.6)	369 (5.3)	988 (6.6)
	Open wound of head		S01	100,554 (8.2)	52,411 (4.4)	955 (13.6)	1198 (8.0)
	Dislocation, sprain and strain—wrist/hand level		S63	41,828 (3.4)	43,519 (3.7)	300 (4.3)	738 (4.9)
	Fracture at wrist and hand level		S62	62,133 (5.0)	47,375 (4.0)	553 (7.9)	1099 (7.3)
	Fracture of forearm		S52	33,021 (2.7)	73,465 (6.2)	523 (7.5)	2541 (16.9)
	Dislocation, sprain and strain—ankle/foot level		S93	61,845 (5.0)	78,686 (6.7)	372 (5.3)	999 (6.6)
	Fracture of shoulder and upper arm		S42	31,012 (2.5)	32,482 (2.7)	636 (9.1)	1585 (10.5)
	Fracture of lower leg, including ankle		S82	31,788 (2.6)	44,951 (3.8)	413 (5.9)	993 (6.6)
	Fracture of foot and toe, except ankle		S92	34,430 (2.8)	44,003 (3.7)	268 (3.8)	816 (5.4)
	Fracture of femur		S72	9562 (0.8)	11,628 (1.0)	306 (4.4)	555 (3.7)
**Multi-mobidity**	*N* diagnoses < 1 year from index*			0.0 (1.0)	0.0 (1.0)	1.0 (2.0)	1.0 (2.0)
**Risk factors**	Risk factor: alcohol			38,422 (3.1)	16,092 (1.4)	705 (10.1)	488 (3.2)
**Presciptions/diagnosis proxies**	Drugs for constipation		A06	143,935 (11.7)	155,495 (13.1)	1833 (26.1)	3603 (24.0)
	Antianemic preparations		B03	115,483 (9.4)	158,437 (13.4)	1394 (19.9)	2717 (18.1)
	Vasoprotectives		C05	271,703 (22.0)	326,502 (27.6)	1443 (20.6)	3851 (25.6)
	Antibacterials, systemic use		J01	1,071,952 (86.9)	1,091,411 (92.2)	6413 (91.5)	14,075 (93.6)
	Analgesics		N02	780,557 (63.3)	850,424 (71.9)	5424 (77.4)	12,206 (81.2)
	Other nervous system drugs		N07	114,681 (9.3)	97,357 (8.2)	1032 (14.7)	1563 (10.4)
**Polypharmacy**	*N* presciptions therapeutic subgroup*			9.0 (8.0)	12.0 (8.0)	13.0 (9.0)	13.0 (9.0)
**Osteoporosis risk**	PPIs	1 year f.I	A02BC	202,031 (16.4)	217,002 (18.3)	1773 (25.3)	3739 (24.9)
		2–5 years f.I		137,288 (11.1)	156,658 (13.2)	847 (12.1)	1988 (13.2)
		6–15 years f.I		151,599 (12.3)	165,175 (14.0)	921 (13.1)	2114 (14.1)
		Never		741,995 (60.2)	644,350 (54.5)	3470 (49.5)	7190 (47.8)
**Fall risk**	Thiazides and potassium, combination	1 year f.I	C03AB	71,260 (5.8)	99,326 (8.4)	469 (6.7)	1573 (10.5)
		2–5 years f.I		46,180 (3.7)	64,577 (5.5)	456 (6.5)	1255 (8.3)
		6–15 years f.I		63,903 (5.2)	97,782 (8.3)	736 (10.5)	1858 (12.4)
		Never		1,051,570 (85.3)	921,500 (77.9)	5350 (76.3)	10,345 (68.8)
	Sulfonamides, plain	1 year f.I	C03CA	64,267 (5.2)	63,860 (5.4)	972 (13.9)	1643 (10.9)
		2–5 years f.I		25,545 (2.1)	27,995 (2.4)	356 (5.1)	558 (3.7)
		6–15 years f.I		22,926 (1.9)	34,228 (2.9)	262 (3.7)	609 (4.1)
		Never		1,120,175 (90.9)	1,057,102 (89.3)	5421 (77.3)	12,221 (81.3)
	ARBs, plain	1 year f.I	C09CA	178,580 (14.5)	164,290 (13.9)	1183 (16.9)	2685 (17.9)
		2–5 years f.I		33,059 (2.7)	31,424 (2.7)	287 (4.1)	563 (3.7)
		6–15 years f.I		35,683 (2.9)	35,201 (3.0)	342 (4.9)	649 (4.3)
		Never		985,591 (79.9)	952,270 (80.5)	5199 (74.2)	11,134 (74.1)
	ARBs and diuretics	1 year f.I	C09DA	45,808 (3.7)	39,887 (3.4)	265 (3.8)	597 (4.0)
		2–5 years f.I		33,786 (2.7)	32,113 (2.7)	253 (3.6)	533 (3.5)
		6–15 years f.I		26,816 (2.2)	26,699 (2.3)	271 (3.9)	608 (4.0)
		Never		1,126,503 (91.4)	1,084,486 (91.7)	6222 (88.7)	13,293 (88.4)
	SSRIs	1 year f.I	N06AB	50,970 (4.1)	83,352 (7.0)	650 (9.3)	1680 (11.2)
		2–5 years f.I		28,511 (2.3)	38,066 (3.2)	236 (3.4)	549 (3.7)
		6–15 years f.I		78,463 (6.4)	109,306 (9.2)	510 (7.3)	1292 (8.6)
		Never		1,074,969 (87.2)	952,461 (80.5)	5615 (80.1)	11,510 (76.6)
	Other ADs	1 year f.I	N06AX	45,582 (3.7)	67,502 (5.7)	506 (7.2)	1396 (9.3)
		2–5 years f.I		28,990 (2.4)	38,113 (3.2)	255 (3.6)	584 (3.9)
		6–15 years f.I		60,702 (4.9)	76,697 (6.5)	401 (5.7)	919 (6.1)
		Never		1,097,639 (89.0)	1,000,873 (84.6)	5849 (83.4)	12,132 (80.7)

*Numeric variables are presented as median (IQR). Other categorical variables are presented as *N* (%). For risk medication, the number of individuals with a last redemptions in the windows 1 year, 2–5 years and 6–15 years f. I. are presented. Abbreviations: *MOF* major osteoporotic fractures, *PPIs* proton pump inhibitors, *ARBs* angiotensin 2-receptor blockers, *f.I* from index, *SSRIs* selective serotonin-reuptake inhibitors, *ADs* antidepressants

### Major osteoporotic fractures

**Fig. 1:**
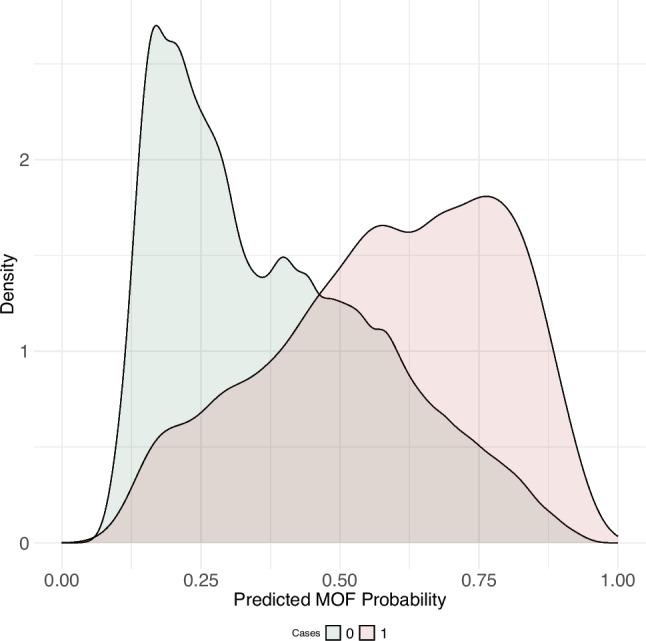
Density plot for major osteoporotic fracture probability across registered cases in the testing sample. The two density plots show the distribution of estimated probabilities between 0 and 1 across individuals who experienced a fracture in the index year (red) or did not (green) Abbreviations: MOF, major osteoporotic fracture

The primary model utilized a total of 2,000 trees using 478 of the 1,900 features provided (Supplementary Table S10). An AUC (95%-CI) of 0.77 (0.76, 0.77) (Supplementary Fig. S1) was determined for the testing cohort. Initially, a Youden’s threshold at an estimated probability of 0.49 maximized the model’s classification sensitivity and specificity (Supplementary Fig. S1). A density plot presenting estimated MOF probabilities across registered MOF fractures showed a clear potential for classification (Fig. 1).

**Fig. 2:**
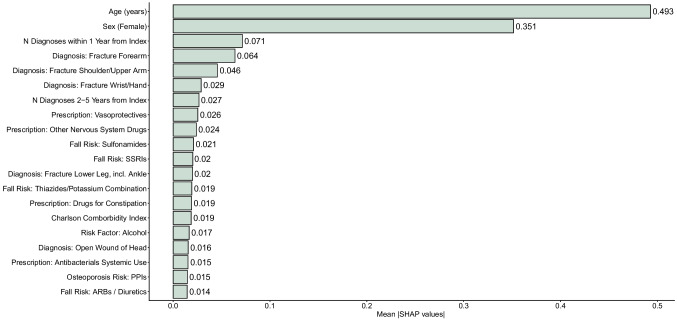
Barplots presenting covariate importance for the 20 most important features in FREM_ML_ using mean |SHAP| values in the testing sample. Abbreviations: SHAP, Shapley Additive exPlanations; *N*, number; SSRIs, selective serotonin receptor inhibitors; ARBs, angiotensin receptor blockers

The mean absolute SHAP values (|SHAP|), representing feature importance, for the twenty most relevant features are presented in Fig. 2. Age and female sex were the strongest predictors of estimated MOF probability, with mean |SHAP| values of 0.49 and 0.35, respectively. In Supplementary Table S10, the mean |SHAP| values for all 478 included features are presented. In Supplementary Fig. S2, a Sina plot demonstrating directions of associations in the testing cohort is presented.

Briefly, the number of registered hospital diagnoses within one year (3rd strongest predictor) and two to five years (7th strongest predictor) were positively associated with predicted MOF risk. Next, previous forearm, shoulder and upper arm, and wrist and hand fractures significantly contributed to estimated MOF risk. SHAP dynamics of the alcohol use risk factor (16th strongest predictor) in some individuals resembled those of a previous wrist or hand fracture (6th strongest predictor), considering both direction and magnitude of association in Supplementary Fig. S2.

### High-risk threshold exploration

In the testing cohort, simply employing the Youden’s threshold would result in a sensitivity of 69.2% while maintaining a specificity of 71.1%, resulting in an accuracy of 71.1%. However, this threshold does not take a feasibility preference towards specificity into account, and does not correct for performance differences across age and sex. Potential thresholds for high-risk classifications were assessed in 5% increments of estimated MOF risk, reporting classification analytics, such as accuracy, sensitivity, specificity, as well as the number of individuals identified as high-risk, MOF events, osteoporosis diagnosis or AOM, and DXA assessments in 2022, Supplementary Table S9.

In the 11,809 and 4,994 individuals who underwent a DXA scan or received a primary osteoporosis diagnosis or medication in 2022 in the testing sample, respectively, median predicted risk estimates by FREM_ML_ (IQR) corresponded to 0.51 (0.28) and 0.58 (0.27).

**Fig. 3:**
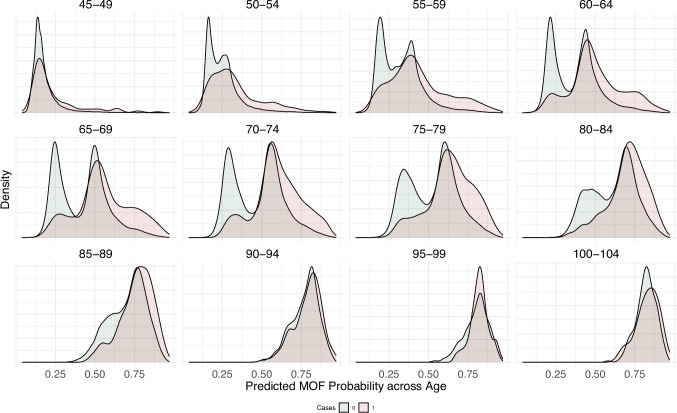
Density plots for sample distribution across predicted risk of MOF and categories of age in the testing sample. Abbrevations: MOF, major osteoporotic fracture

In Supplementary Fig. S3, density plots for relative risk of MOF sex-adjusted risk estimates, age-adjusted risk estimates, and age- and sex-adjusted risk estimates are presented. Subgroup analyses were performed by age and sex. Estimated MOF probabilities stratified across five-year increments of age are presented in Fig. 3. Moreover, estimated probabilities extracted from the non-stratified models differed substantially across sex, as shown in Supplementary Fig. S4. When the Youden’s index was computed in the testing sample stratified by sex, the threshold maximizing sensitivity and specificity was reported at 0.59 in women and 0.39 in men.

In response, optimised thresholds for each age- and sex-specific category were calculated using the Youden’s index in the training and validation sample, Supplementary Table S11. These cutoffs were also presented as relative risk estimates compared to the mean estimated risk in each category to highlight the specific degree of risk increases, considering the average risk in the population. Across the complete testing population, age- and sex-adjusted thresholds resulted in a sensitivity of 51.3%, specificity of 73.4%, and an accuracy of 73.2%, Supplementary Table S12. While the accuracy would be superior to using a single cutoff, the sensitivity of age- and sex-specifc Youden’s cutoffs was markedly reduced using a Youden’s approach, while reducing the number of false positive classifications by 2.3%.

To promote feasibility, thresholds supporting a specificity of 80% in each age and sex category were also explored, Supplementary Table S12, resulting in a sensitivity, specificity, and accuracy of 40.4%, 79.9%, and 79.6% in the testing cohort, respectively.

### Sex-stratified models

In women, a stratified model presented an AUC (95%CI) of 0.73 (0.72, 0.74) in the testing cohort. Furthermore, a threshold of 0.45 for MOF classification was determined using Youden’s index, Supplementary Fig. S5. At this cut-off, a sensitivity of 0.62 (PPV 0.027) at a specificity of 0.72 (NPV 0.993) was reached at an overall accuracy of 0.72.

The stratified model in men achieved an AUC (95%CI) of 0.75 (0.73, 0.76). A threshold of 0.48 was determined to achieve the best classification sensitivity and specificity in males, Supplementary Fig. S5. At this cut-off, an accuracy of 76.4% was reached at sensitivity of 0.63 (PPV 0.015) and a specificity of 0.76 (NPV 0.997). Distributions of estimated probabilities across MOF status are presented in Supplementary Fig. S6. In total, 412 and 378 features were included in the model in women and men, respectively, and the most relevant features in women and men are presented in Supplementary Figs. S7 and S8, respectively. In summary, the non-stratified model outperformed sex-stratified models, considering sensitivity and specificity.

### Hip fractures

Despite the large study population, the number of hip fractures registered within a one-year index period was relatively small, limiting the potential to facilitate the development of a reliable ML model. Therefore, HF prediction was only explored in secondary analyses. During model evaluation, an AUC (95% CI) of 0.85 (0.85, 0.86) was calculated, and a sensitivity and specificity were maximized at a classification cutoff of 0.72, Supplementary Fig. S9. When the estimated probabilities were graphed across HF event status, an asymmetric multi-centered distribution was observed, Supplementary Fig. S9, suggesting weak model performance for classification of HF risk in clinical care.

## Discussion

Overall, FREM_ML_ model evaluation demonstrated an AUC of 0.77 and suggested superiority over FREM_ver1_ and FREM_ver2_ approaches, employing logistic regression and fewer predicting variables [11, 14]. Indeed, the improved performance highlights that the gradient boosting model may recognize non-linear patterns present in the FREM cohort. Subgroup analyses indicated that accurate classification of imminent risk is possible, when age- and sex-adjusted relative risk estimates are used. Next, the AUC indicates that the proposed model is not perfect, which may be attributed to the nature of the data itself and the deliberate effort to prevent overfitting. While FREM_ML_ has a higher predictive power, it is not as easily interpreted as older versions. However, by adding SHAP values to personalized model outputs, corresponding insights may be used to motivate and justify clinical decision making. Linked with risk factors and reference risk estimates stratified for age and sex, the FREM_ML_ approach could facilitate the development of an accurate and robust decision-support tool for general practitioners (GPs) in primary care.

### Integration in current literature

In contrast to other available risk prediction tools models, FREM_ML_ will be shared a publicly available format with full transparency, in accordance with the European AI act for High Risk AI Systems [28]. Indeed, as this decision-support tool is solely intended to be used for the prescription of DXA diagnostic assessments, there is no direct risk of bodily harm resulting from limitations of specificity, such as for models assigning pharmacological interventions. This contrasts the FREM_ML_ methodology from the FRAX® algorithm, which, in some countries, is used for the direct prescription of anti-osteoporotic medication without a previous DXA scan [29].

Due to the high quality of Danish national register data, the FREM_ML_ does not rely on any manual data input nor is it subject to recall bias. Upon implementation, it could therefore be easily integrated into electronic medical journals, allowing for automatic risk predictions re-calculated upon every visit to the GP. This was one motivation for using a one-year risk window to allow for a more imminent and dynamic risk profiling. Essentially, this will highlight the need for referral for DXA in patients who have not already been diagnosed with osteoporosis on an annual basis.

Briefly, we observed reduced model performance in men. This may be explained by smaller MOF event rates, which increases sample imbalance and may result in overfitting of the non-stratified model to the female population. Concerning ICD-10-based diagnoses and medication, men tend to seek health care less commonly than women, reducing the number of available predictors for MOF in registry data [30]. Furthermore, age may be a less granular predictor in men compared to women, due to a reduced life expectancy and a higher competing risk of mortality. Importantly, adding a proxy for alcohol use, extracted solely from registry data contributed strongly to the prediction performance in males.

A comparable fully automated model proposed is Crystal Bone [15]. With an overall AUC of 0.81 and a one- to two-year risk prediction, this temporal deep-learning algorithm seems promising in the testing cohort of approximately 200,000 individuals. However, the generalizability of a long short-term memory model trained on eHRs from the United States may be limited concerning the Danish health services, and Crystal Bone is a proprietary software. Therefore, future efforts of the FREM_ML_ approach strive to explore natural language-based algorithms and rolling exposure windows in a Danish context.

As most prediction models describe AUCs of approximately 0.8 [15, 31], this could indicate that it may be the maximal model performance possible with eHRs. As FREM_ML_ supplies clinicians with personalized risk profiles compiled using SHAP values, we make a seemingly “imperfect” model clinically relevant by combining human and artificial intelligence. Thereby, required information for referral to DXA assessments is compressed to effectively identify individuals at imminent fracture risk.

### Future outlook and clinical implementation

Importantly, a recent perspective on the use of artificial intelligence in fracture prediction [18] suggested that the clinical integration of ML algorithms may transform osteoporosis care in an aging population. In principle, this study indicates the initial clinical potential of FREM_ML_ by facilitating the interpretation of risk predictions using XAI. If implemented optimally in primary care, a decision-support system might provide GPs with detailed insights into their patients’ personalized fracture risk profile, extracted from SHAP estimates. The approach is intended to improve integration into the clinical pathway by offering not only a risk estimate but also pointing out to clinicians which features of a patient medical history has contributed to a high imminent risk classification. This may be important in justifying the decision to offer treatment in terms of guidelines, patient information, and informed choice. Moreover, according to the economic costs of fractures, implementing DXA scans in individuals at high FREM_ML_-predicted risk of MOF might be economically beneficial, even if may just prevent a few MOF events [32]. This should be shown in additional validation and implementation studies [33].

**Fig. 4:**
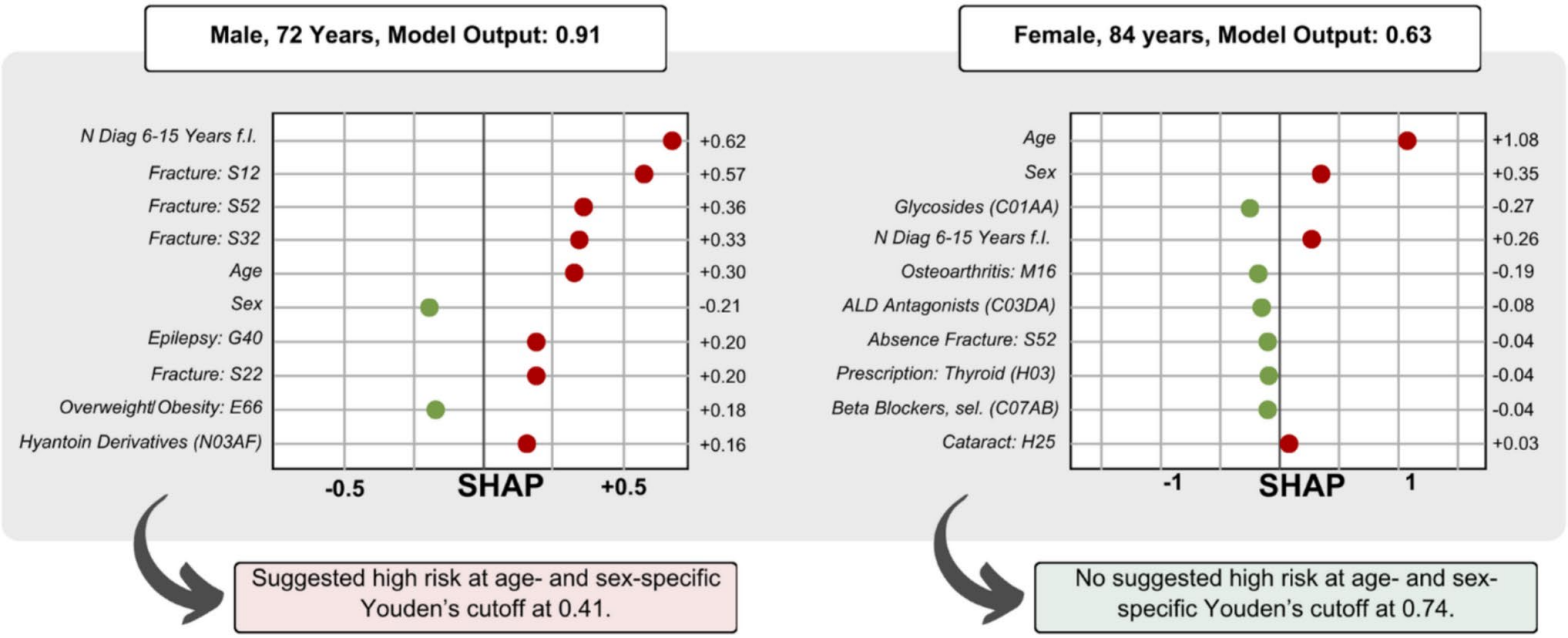
Case examples FREM-ML explainable AI outputs. Abbreviations: *N*, number; SHAP, Shapley Additive exPlanations; ALD, aldosterone receptor; sel., selective. For ICD-10 codes, see International Classifications of Diseases

We highlight this potential in two case examples. First, personalized risk predictions were computed using FREM_ML_, and age- and sex-specific Youden’s cutoffs were obtained. (Supplementary Table S11). To analyse feature importance in these individuals, personalized SHAP values could be extracted – highlighting the impact of the ten most important predictors of risk in an individual. In Fig. 4, we present two hypothetical case examples, which are further explained in Supplementary Method Sect. 1: “Explainable AI and the FREM_ML_ Algorithm explained for general practitioners without experience in artificial intelligence”.

In future projects, we aim to calibrate and validate FREM_ML_ further. For instance, we will explore a harmonized data model across Denmark, Sweden, and Norway [34] and define optimal national thresholds for relative estimated MOF risk to motivate DXA-based osteoporosis screening using FREM_ML_, employing a recently collected dataset of available BMD assessments in Denmark. For implementation feasibility in primary care, an appropriate balance between specificity and sensitivity must be met. Moreover, the feasibility of explainable AI in a clinical setting, such as SHAP values and predicted relative risk based on reference values, must be optimized further using qualitative studies including all stakeholders. Lastly, model performance may be further improved by integrating other features, such as wearable-based data, into eHRs to obtain insights on lifestyle and gait features, in addition to more high-quality data from primary care.

### Strengths and limitations

The development of FREM_ML_ has several strengths and some limitations. For instance, the model was trained and validated in a large sample, including high-quality data, in all Danish individual aged 45 years and older [19–21, 23]. As no manual data input is required, this reduces recall and social desirability bias. The employed algorithm is a powerful prediction tool, able to account for non-linear trends, small unique subgroups of individuals, and personal risk profiles. Next, subgroup and secondary analyses provided insights into the next steps required for clinical implementation. However, fractures are difficult events to predict as they are affected by randomness introduced by random accidents. Therefore, a perfect AUC is not possible. In the future, the FREM_ML_ model still requires validation and calibration to optimize thresholds for osteoporosis case finding. Furthermore, a randomized controlled trial must be performed in general practice, integrating the approach into clinical workflows. Next, there may be some characteristics not measured in the eHRs, such as physical activity and granular information on alcohol use, which substantially contribute to fracture risk. In the future, an addition of wearable data should be explored to automatically obtain gait characteristics and automatic activity assessments in general practice. Finally, as this model was trained on Danish data, its generalizability to other populations requires validation and calibration in other countries, with upcoming studies scheduled in Sweden and Norway [34].

## Conclusion

In conclusion, FREM_ML_ enhanced the latest available FREM decision-support approach by leveraging artificial intelligence in the form of a boosting model. By integrating age- and sex-based thresholds for DXA assessments, the FREM_ML_ decision-support system may aid future primary detection of osteoporosis in primary care and the prevention of fractures in an aging population.

## Supplementary Information

Below is the link to the electronic supplementary material.

ESM 1 DOCX 3.62 MB

## Data Availability

FREM_ML_ was trained in Danish national Registry data available upon a formal request. To protect patient confidentiality, the complete dataset can only be accessed through an authorized intermediary, the Danish Health Data Authority, in accordance with Danish data regulations. For requests, please contact them via their official website: https://english.sundhedsdatastyrelsen.dk/.
