# Supplementary material for: Introducing FREMML: a decision-support approach for automated identification of individuals at high imminent fracture risk
Source: Arch Osteoporos. 2025 Nov 5;20(1):140. doi: 10.1007/s11657-025-01613-5 (PMC12589358; doi:10.1007/s11657-025-01613-5)
Supplement: Supplementary file 1 — DOCX 3.62 MB [file 11657_2025_1613_MOESM1_ESM.docx]

**Supplementary Material**

Introducing FREMML: A Decision-support Approach for Automated Identification of Individuals at High Imminent Fracture Risk

Authors:

Marlene Rietz ^1-3^, Jan C. Brønd ^1,4^, Sören Möller ^5^, Jens Søndergaard ^6^, Bo Abrahamsen ^1,4^, Katrine Hass Rubin ^1,4^

*^1^ Research Unit OPEN, Department of Clinical Research, University of Southern Denmark, Odense, Denmark.*

*^2^ Steno Diabetes Center Odense, Department of Clinical Research, University of Southern Denmark, Odense, Denmark.*

*^3^ Division of Clinical Physiology, Department of Laboratory Medicine, Karolinska Institutet, Huddinge, Sweden.*

*^4^ OPEN - Open Patient data Explorative Network, Odense University Hospital, Odense, Denmark.*

*^5^ Research Unit for Epidemiology, Biostatistics and Biodemography, Department of Public Health, University of Southern Denmark, Odense, Denmark*

*^6^ Research Unit for General Practice, Department of Public Health, University of Southern Denmark, Odense-Esbjerg*

Corresponding author:

Katrine Hass Rubin^a, b^

E-mail address: Katrine.Rubin@rsyd.dk

Telephone: +45 21261966

**Supplementary Method Section**

**Supplementary Method Section 1.** Explainable AI and the FREM_ML_ Algorithm explained for general practitioners without experience in artificial intelligence.

**Supplementary Method Section 2.** TRIPOD-AI Checklist

**Supplementary Tables**

**Supplementary Table S1** – Exclusion Criteria Osteoporosis and Osteoporotic Fractures

**Supplementary Table S2** – Diagnosis Feature Engineering

**Supplementary Table S3** – Definition of Diagnosis-based Risk Factors

**Supplementary Table S4** – N with assigned Risk Factors (%) by Risk Factors

**Supplementary Table S5** – Medication Feature Engineering

**Supplementary Table S6** – Medication N Redemptions (%) by Medication Feature

**Supplementary Table S7** – Comparison of Training and Testing Sample

**Supplementary Table S8** – Hyperparameter Search Space and Selections

**Supplementary Table S9** – Model Analytics by Probability Thresholds for Major Osteoporotic Fractures in the Testing Sample

**Supplementary Table S10** – Features included in the Model for Major Osteoporotic Fractures

**Supplementary Table S11** – Age- and sex-specific cutoffs including model analytics in the Testing Sample

**Supplementary Figures**

**Supplementary Figure S1** – ROC Curve and Accuracy by Probability Cutoff for the MOF-prediction model in all individuals > 45 years.

**Supplementary Figure S2** – Sina plot of SHAP values for the 20 most important features included in the non-stratified major osteoporotic fracture prediction model in the testing sample

**Supplementary Figure S3** – Density plots for sample distribution across predicted risk of MOF and sex in the complete sample showing the fixed non-stratified Youden’s threshold (red) and a sex-specific threshold (grey).

**Supplementary Figure S4** – ROC Curves for and Accuracy by Probability Cutoff for the MOF-prediction model in women (red) and men (blue) >45 years

**Supplementary Figure S5** – Density Plot for Major Osteoporotic Fracture Probability across registered Cases in models trained separately in women (red) and men (blue).

**Supplementary Figure S6** – Barplots for mean SHAP values for the 20 most important features included in the major osteoporotic fracture model in women.

**Supplementary Figure S7** – Barplots for mean SHAP values for the 20 most important features included in the major osteoporotic fracture model in men.

**Supplementary Figure S8** – Density Plot for Relative Risk of MOF sex-adjusted Risk Estimates, age-adjusted Risk Estimates, and age- and sex-adjusted Risk Estimates.

**Supplementary Figure S9** – ROC Curves and Accuracy by Probability Cutoff for the Hip Fracture prediction model, as well as a Density Plot for Hip Fracture Probability across registered Case.

**Supplementary Method Section 1: Explainable AI and the FREM_ML_ Algorithm explained for General Practitioners without Experience in Artificial Intelligence.**

**What is Explainable AI?**

Gradient-boosting models can be considered explainable black-box models, a term that refers to the contrast between traditional and transparent white box models, such as logistic regression, and the black box limitation in machine learning algorithms. The black box phenomenon in disease risk prediction describes ambiguous decision making processes hidden within complex algorithms, which result in the inability of interpreting model structures.^1^ Therefore, questions by patients or insurance companies on why a specific intervention has been assigned may not be answered directly. For instance, black box limitations occur when the multi-dimensional model architecture of a machine learning algorithm is too complex to interpret, or when proprietary algorithms are not shared publicly.^2^ However, considering proprietary algorithms, even outputs of traditional white box models (such as FRAX) may be ambiguous if the algorithm components or weights are not open source.^3^

While most machine learning algorithms provide superior predictions and classifications compared to traditional statistical models, model outputs need to be explained if considered within clinical care.

The term explainable AI, referring to our gradient-boosting model, highlights that risk prediction outputs are backed by SHapley Additive exPlanations (SHAP) values presenting the contribution of different features to the model output on an individual level.^4^ Next, there is an important human component to explainable AI. Briefly, a report by the European Data Protection Supervisor (EDPS) summarizes that to implement and trust AI, provided interpretation tools must be understandable, contrastive, and contextual.^5^ Furthermore, clinicians judging model outputs are subjective and affected by social norms and morals concerning the application of AI.

*
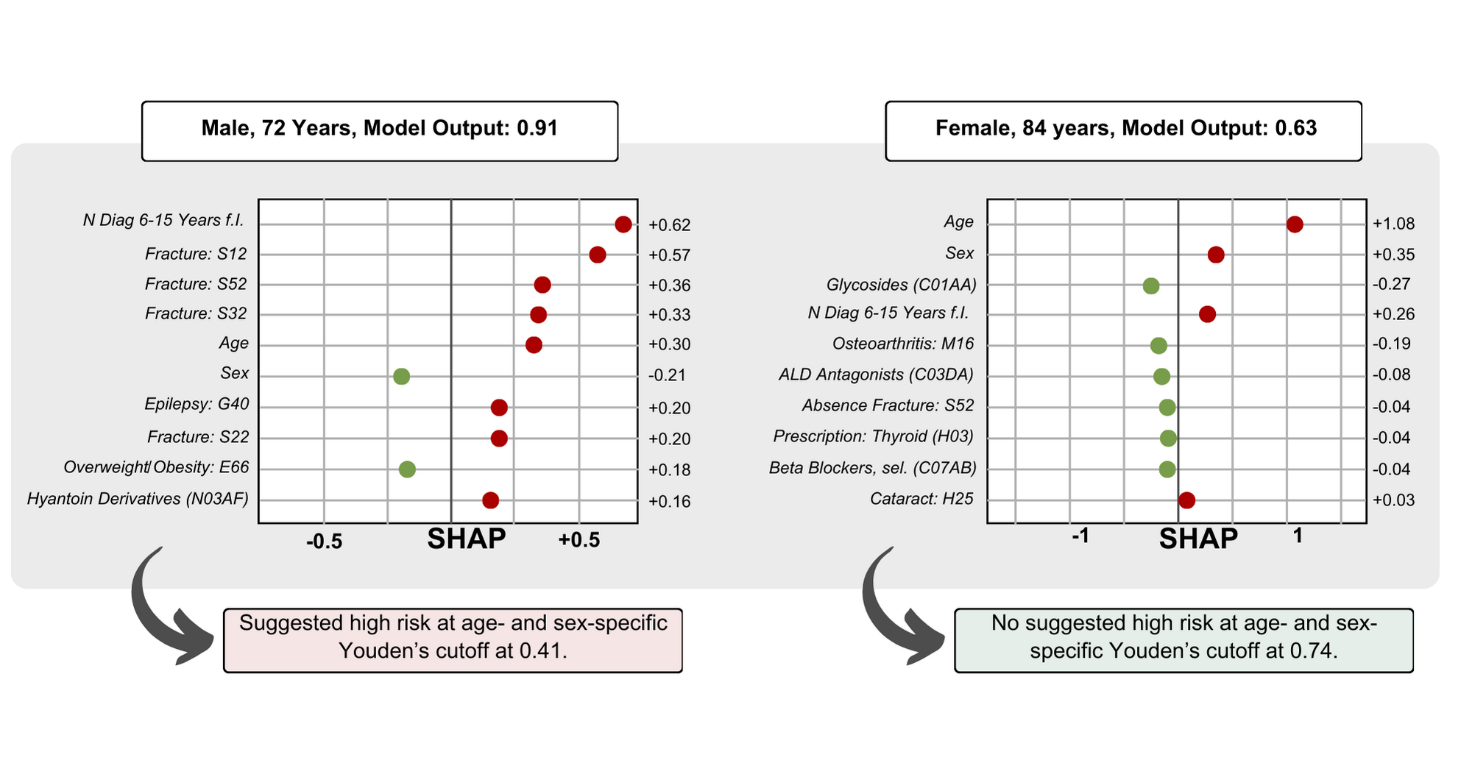
*

**Fig 4**. Case Examples FREM-ML Explainable AI Outputs. | *Abbreviations: N – number, SHAP – Shapley Additive exPlanations, ALD – aldosterone receptor; sel. - selective. For ICD-10 codes see International Classifications of Diseases*

In the following, we will demonstrate the potential of SHAP values for advancing personalized endocrinology using a brief description and two clinical cases.

**Implementation of FREM_ML_ in Clinical Care**

We must highlight again that the purpose of FREM_ML_ is not the automatic assignment of pharmacological interventions against fractures, such as in FRAX in some cases, but to support the decision of whether an individual should participate in more detailed screening for osteoporosis, such as a dual x-ray absorptiometry (DXA) scan. For instance, FREM_ML_ could be implemented in Danish electronic health records, and at every visit to the general practitioner (GP), a FREM estimate for diagnostic testing of osteoporosis can be recommended to the GP. First, the patient’s medical information would be fed into the FREM_ML_ algorithm, which supplies a raw risk estimate between 0-1 as well as SHAP values for all features. Next, the individual would be classified as high risk candidate, if the estimated risk is above a certain age- and sex-specific threshold. Then, easily understandable SHAP values can be graphed in a Sina plot, presenting the ten most important features in an individual, and the relative risk is shown on a plot with intervention referral thresholds by age and sex. Finally, the GP may interpret the certainty of the risk estimate, and make a manual decision on whether an individual should undergo a DXA scan.

**Case Example 1: Identifying Individuals at High Risk of MOF**

Let us imagine that a 72-year old men, a new patient, comes to a primary care center for a health check. From FREM_ML,_ a MOF risk of 0.91 is estimated, i.e., the risk of MOF is significantly elevated compared to the age- and sex-specific cutoff at 0.41. In the SHAP plot (Figure 4), we observe that the most important feature in the man is the number of diagnoses from 6 to 15 years ago, suggesting chronic diseases. We even see the risk direction, i.e. risk increase or decrease, of each relevant feature. Next, we see that the risk estimate is elevated due to previous fractures of the neck / thorax (S12), forearm (S52), lumbar spine / pelvis (S32), and ribs / sternum (S22). Furthermore, the individual suffers from epilepsy (G40), has been diagnosed with overweight or obesity (E66), and redeemed the fall risk medication hyantoin derivatives (N03AF). Based on the explainable output, we go into the patient’s medical records, check for previous DXA scan results and see that the fractures occurred isolated throughout the 15-year lookback period. On the age- and sex-stratified intervention threshold, the individual is classified as high risk, warranting a DXA scan (cutoff at 0.41). Finally, we agree with the fracture risk evaluation and risk estimate of FREM_ML_, and assign a DXA scan.

**Case Example 2: Uncertainty in FREM_ML_ due to low-risk profile in old age**

On a group level, the strongest contributors to the FREM_ML_ model output are age and sex. Therefore, the mere combination of old age and female sex is associated with a substantial risk increase in an individual, even in absence of other risk factors. As these factors are non-modifiable, the primary features of interest entail previous medication, diagnoses, and risk factors of MOF.

For instance, a 84 year-old woman comes to the GP and is automatically assigned a FREM_ML_ predicted MOF risk of 0.63, i.e., a risk estimate below an age- and sex-specific cutoff of 0.74 for high MOF risk. In the SHAP plot (Figure 4), we see that the risk prediction is at risk of uncertainty as risk estimates rely majorly on the individual’s age and sex. In detail, the SHAP value for age is almost two times as high as the most important predictor in Case 1. The woman’s risk lowered by an absence of a previous S52 fracture. Relevant diagnoses were coxathrosis (M16) and age-related cataract (H25) in the model. ATC codes affecting model output are fall-risk relevant cardiac glycosides (C01AA), aldosterone antagonists (C03DA), beta blockers (C07AB), and redemption of thyroid hormones (H03) medication. Interestingly, the diagnoses of osteoarthritis as well as the prescription of cardiac glycosides and aldosterone antagonists are included as risk lowering features. After understanding this output, the GP can then make an informed decision on whether an age- and/or sex-motivated DXA scan is required.

**References**

1. Ali, S., Abuhmed, T., El-Sappagh, S., Muhammad, K., Alonso-Moral, J. M., Confalonieri, R., et al. (2023). Explainable Artificial Intelligence (XAI): What we know and what is left to attain Trustworthy Artificial Intelligence. Information Fusion, 99, Article 101805. https://doi.org/10.1016/j.inffus.2023.101805 [storage.prod.researchhub.com+2scirp.org+2](https://storage.prod.researchhub.com/uploads/papers/2024/02/28/1-s2.0-S1566253523001148-main.pdf?utm_source=chatgpt.com)
2. Xu, H., & Shuttleworth, K. M. J. (2024). Medical artificial intelligence and the black box problem: A view based on the ethical principle of “do no harm”. Intelligent Medicine, 04, 52–57. https://doi.org/10.1016/j.imed.2023.08.001
3. Allbritton-King, J. D., Elrod, J. K., Rosenberg, P. S., & Bhattacharyya, T. (2022). Reverse engineering the FRAX algorithm: Clinical insights and systematic analysis of fracture risk. Bone, 159, 116376. https://doi.org/10.1016/j.bone.2022.116376
4. Lundberg, S. M., & Lee, S.-I. (2017). A unified approach to interpreting model predictions. In Advances in Neural Information Processing Systems, 30 (pp. ???–???) [or “(NeurIPS 2017)”]. (Available as arXiv preprint: arXiv:1705.07874) [aepd.es+3Scott Lundberg+3arXiv+3](https://scottlundberg.com/publication/shap_nips/?utm_source=chatgpt.com)
5. European Data Protection Supervisor. (2023). EDPS TechDispatch on Explainable Artificial Intelligence. [https://www.aepd.es/documento/techdispatch-xai.pdf](https://www.aepd.es/documento/techdispatch-xai.pdf?utm_source=chatgpt.com)

**Supplementary Method Section 2: TRIPOD-AI Checklist**


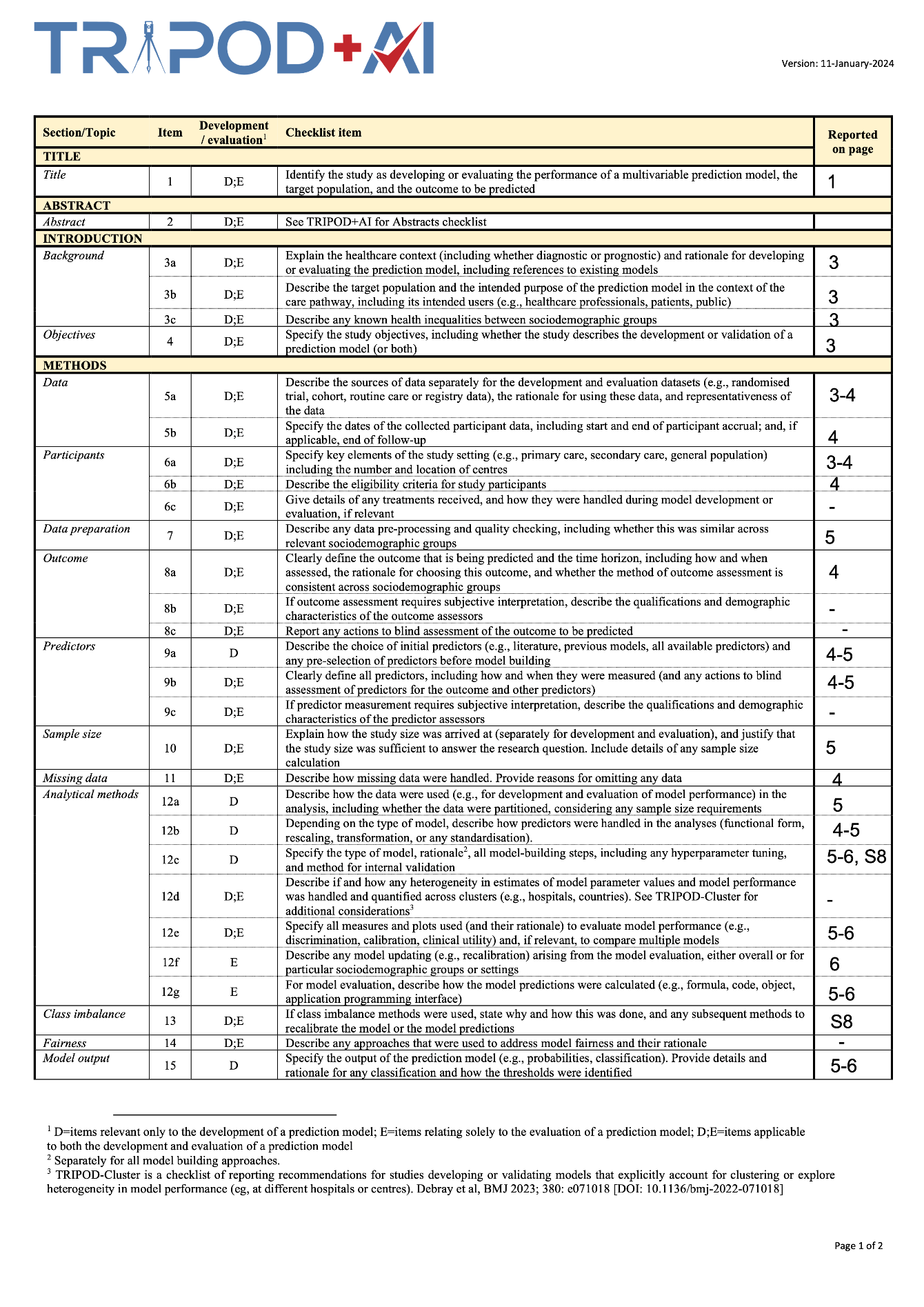


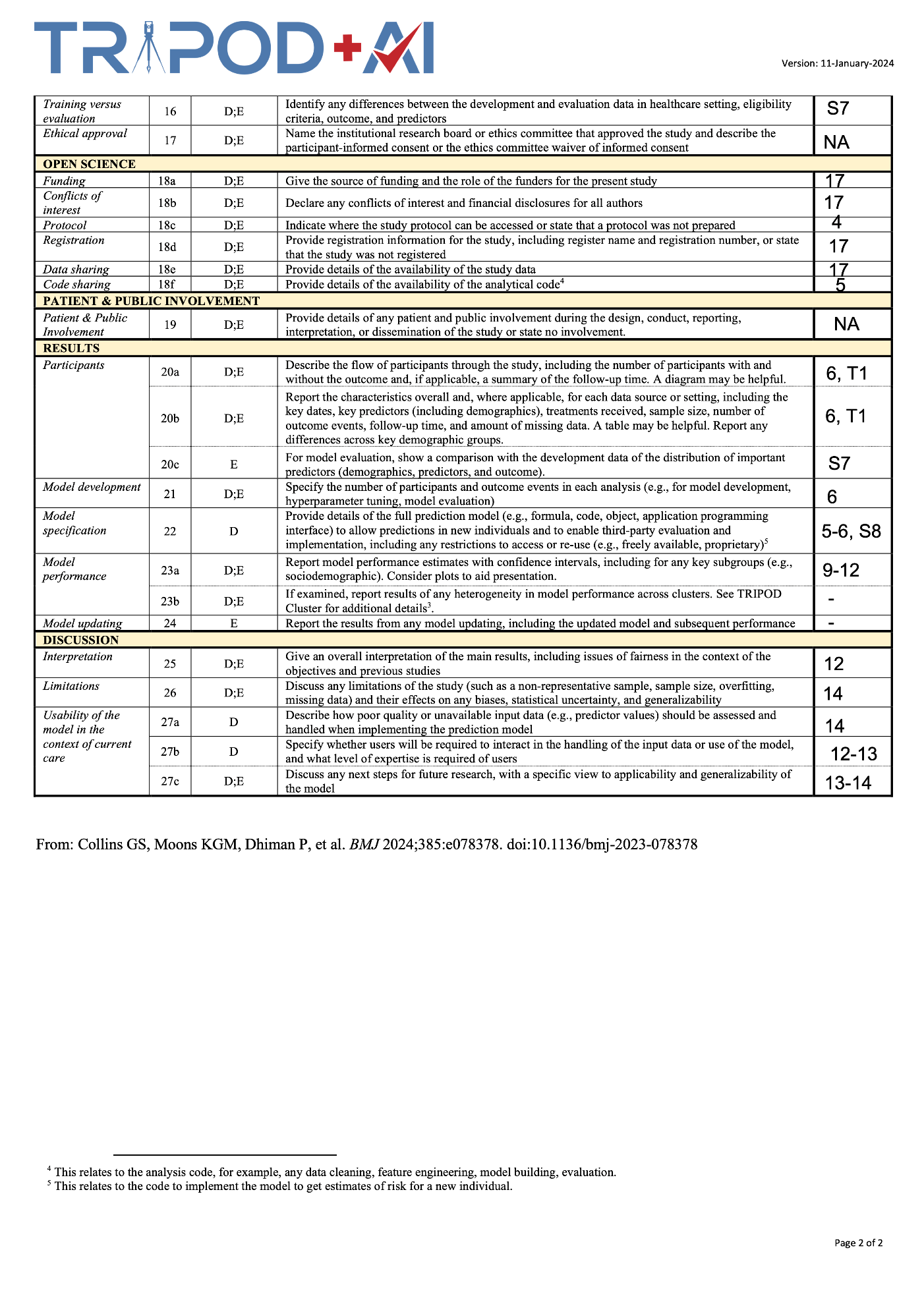


**Supplementary Table 1. Exclusion Criteria Osteoporosis and Osteoporotic Fractures**

| Name | ATC code (DNPR) | Procedures used in the Danish hospitals | ICD-10 code |
| --- | --- | --- | --- |
| Osteoporosis treatment | | | |
| Bisphosphonate | M05BA01, M05BA04, M05BA06, M05BA07, M05BA08, M05BB01, M05BB03 | BWHB40A, BWHB40B |  |
| Strotium ranelate | M05BX03 |  |  |
| Denosumab | M05BX04 | BWHB42 |  |
| Romozosumab | M05BX06 |  |  |
| Teriparatide | H05AA02 |  |  |
| Raloxifen | G03XC01 |  |  |
| Osteoporosis diagnosis | | | |
|  |  |  | M80, M81 |

**Supplementary Table 2. Diagnosis Feature Engineering**

| Definition | ICD-10 | Exposure | Format |
| --- | --- | --- | --- |
| Diagnoses | Category (e.g., C09) | 15 years | Binary (any diagnosis) |
| Risk Factors | see Supplementary Table 3 | 15 years | Days from first diagnosis |
| Multimorbidity – 1y | Alpha (e.g. C) | 1 year | Count (unique alpha ICD-10 codes) |
| Multimorbidity – 2-5 y | Alpha (e.g. C) | year 2 – year 5 | Count (unique alpha ICD-10 codes) |
| Multimorbidity – 6-15 y | Alpha (e.g. C) | year 6 – year 15 | Count (unique alpha ICD-10 codes) |
| Charlson Comorbidity Index |  | 15 years | Numeric (score) |

**Supplementary Table 3. Definition of Diagnosis-based Risk Factors**

| Charlson Comorbidities | ICD10 Codes |
| --- | --- |
| Congestive Heart Failure | I09.9, I11.0, I13.0, I13.2, I25.5, I42.0, I42.5-I42.9, I43.x, I50.x, P29.0 |
| Dementia | F00.x-F03.x, F05.1, G30.x, G31.1 |
| Chronic Pulmonary Disease | I27.8, I27.9, J40.x-J47.x, J60.x-J67.x, J68.4, J70.1, J70.3 |
| Rheumatologic Disease | M05.x, M06.x, M31.5, M32.x-M34.x, M35.1, M35.3, M36.0 |
| - Polymyalgia Rheumatica | M35.3 |
| - Rheumatoid Arthritis | M06.9 |
| Mild Liver Disease | B18.x, K70.0-K70.3, K70.9, K71.3-K71.5, K71.7, K73.x, K74.x, K76.0, K76.2-K76.4, K76.8, K76.9, Z94.4 |
| Moderate to severe liver disease | I85.0, I85.9, I86.4, I98.2, K70.4, K71.1, K72.1, K72.9, K76.5, K76.6, K76.7 |
| Diabetes with chronic complications | E10.0, E10.1, E10.6, E10.8, E10.9, E11.0, E11.1, E11.6, E11.8, E11.9, E12.0, E12.1, E12.6, E12.8, E12.9, E13.0, E13.1, E13.6, E13.8, E13.9, E14.0, E14.1, E14.6, E14.8, E14.9 |
| Hemiplegia or Paraplegia | G04.1, G11.4, G80.1, G80.2, G81.x, G82.x, G83.0-G83.4, G83.9 |
| Renal Disease | I12.0, I13.1, N03.2-N03.7, N05.2-N05.7, N18.x, N19.x, N25.0, Z49.0-Z49.2, Z94.0, Z99.2 |
| Any malignancy (including leukemia and lymphoma) | C00.x-C26.x, C30.x-C34.x, C37.x-C41.x, C43.x, C45.x-C58.x, C60.x-C76.x, C81.x-C85.x, C88.x, C90.x-C97.x |
| Metastatic Solid Tumor | C77.x-C80.x |
| AIDS / HIV | B20.x-B22.x, B24.x |
| Other Comorbidities | ICD10 Codes |
| Anorexia Nervosa | F50 |
| Malabsorption | K90 |
| Primary Hyperparathyroidism | E210 |
| Hyperthyreoidism | E05, E06 |
| Cushing's Syndrome | E249, E240, E242, E248 |
| Ankylosing Spondylitis | M459 |
| Mastocytosis | Q822 |
| Multiple Sclerosis | G35 |
| Gout | M100-M104, M109 |
| Diabetes | E10.x-E14.x |
| Osteogenises imperfecta | Q78.0 |
| Early Menopause | N95.1, N95.0, N95.3, E28.3, E89.4, N97.0 |
| Other Risk Factors | ICD10 Codes |
| Anorexia, Underweight, Abnormal Weight Loss | F50.x, E40.x-E47.x, R634.x |
| Lack of vitamins and minerals (D, C, B, calcium, zinc, tiamin, niacin) | E55.x, E58.x, E50.x, E60.x, E54.x, E51.x, E52.x, E53.x |
| Current Smoker | F17.x, Z72.0 |
| Alcohol: Dependency | F10.x |
| Alcohol: Consumption ≥3 units/day | F10.x, K70.x, E52.x, T51.x, K86.0, E24.4, G31.2, I42.6, O35.4, Z71.4, Z72.1, DG62.1, G72.1, K29.2, L27.8A |
| Age-related muscle weakness | R54.x, M63.8 |
| Consultation: Lack of Physical Activity | Z72.3 |
| Consultation: Wrong Nutrition | Z72.4 |
| Consultation: Stress | Z73.x |
| Consultation: Frailty / Need of Help | Z74.x |

**Supplementary Table 4. N with assigned Risk Factors (%) by Risk Factors**

| Risk Factors | N with Risk Factor (%) |
| --- | --- |
| Congestive Heart Failure | 15,762 (0.65) |
| Dementia | 23,699 (0.97) |
| Chronic Pulmonary Disease | 145,924 (5.99) |
| Rheumatologic Disease | 37,454 (1.54) |
| - Polymyalgia Rheumatica | 9,169 (0.38) |
| - Rheumatoid Arthritis | 8,735 (0.36) |
| Mild Liver Disease | 32,119 (1.32) |
| Moderate to severe liver disease | 5,684 (0.23) |
| Diabetes with chronic complications | 140,032 (5.74) |
| Hemiplegia or Paraplegia | 6,820 (0.28) |
| Renal Disease | 32,712 (1.34) |
| Any malignancy (including leukemia and lymphoma) | 195,904 (8.03) |
| Metastatic Solid Tumor | 15,446 (0.63) |
| AIDS / HIV | 1,085 (0.04) |
| Anorexia Nervosa | 534 (0.02) |
| Malabsorption | 5,088 (0.21) |
| Primary Hyperparathyroidism | 8,690 (0.36) |
| Hyperthyreoidism | 45,391 (1.86) |
| Cushing's Syndrome | 643 (0.03) |
| Ankylosing Spondylitis | 4,204 (0.17) |
| Mastocytosis | 399 (0.02) |
| Multiple Sclerosis | 10,167 (0.42) |
| Gout | 24,790 (1.02) |
| Diabetes | 146,437 (6.01) |
| Osteogenises imperfecta | 38 (0) |
| Early Menopause | 18,323 (0.75) |
| Anorexia, Underweight, Abnormal Weight Loss | 13,272 (0.54) |
| Lack of vitamins and minerals (D, C, B, calcium, zinc, tiamin, niacin) | 40,676 (1.67) |
| Current Smoker | 44,309 (1.82) |
| Alcohol: Dependency | 48,763 (2) |
| Alcohol: Consumption ≥3 units/day | 55,707 (2.28) |
| Age-related muscle weakness | 139 (0.01) |
| Consultation: Lack of Physical Activity | 212 (0.01) |
| Consultation: Wrong Nutrition | 850 (0.03) |
| Consultation: Stress | 3,640 (0.15) |
| Consultation: Frailty / Need of Help | 3,262 (0.13) |

**Supplementary Table 5. Medication Feature Engineering**

| Definition | ATC | Exposure | Format | N features |
| --- | --- | --- | --- | --- |
| Osteoporosis Risk Medication | Level 4 | 15 years | Days from last redemption | 16 |
| Fall Risk Medication | Level 4 | 15 years | Days from last redemption | 79 |
| Recent Fall Risk Exposure | Level 4 | 6 months | Binary (any redemption) | 79 |
| Diagnosis Proxies | Level 2 | 15 years | Binary (any redemption) | 90 |
| Poly Medication | Level 1 | 15 years | Count (unique anatomical/pharmacological groups) | 1 |

**Supplementary Table 6. Medication N Redemptions (%) by Medication Feature**

| Feature Type | Name | ATC Codes | N Redemptions (%) |
| --- | --- | --- | --- |
| **Diagnosis Proxies** | Stomatological preparations | A01 | 258,261 (10.59) |
| **Diagnosis Proxies** | Drugs for acid related disorders | A02 | 1,080,160 (44.3) |
| **Diagnosis Proxies** | Drugs for functional gastrointestinal disorders | A03 | 243,661 (9.99) |
| **Diagnosis Proxies** | Antiemetics and antinauseants | A04 | 44,259 (1.82) |
| **Diagnosis Proxies** | Bile and liver therapy | A05 | 1,750 (0.07) |
| **Diagnosis Proxies** | Drugs for constipation | A06 | 304,866 (12.5) |
| **Diagnosis Proxies** | Antidiarrheals, intestinal antiinflammatory/antiinfective agents | A07 | 201,436 (8.26) |
| **Diagnosis Proxies** | Antiobesity preparations, excl. diet products | A08 | 50,068 (2.05) |
| **Diagnosis Proxies** | Digestives, incl. enzymes | A09 | 5,780 (0.24) |
| **Diagnosis Proxies** | Drugs used in diabetes | A10 | 263,460 (10.81) |
| **Diagnosis Proxies** | Vitamins | A11 | 23,003 (0.94) |
| **Diagnosis Proxies** | Mineral supplements | A12 | 298,892 (12.26) |
| **Diagnosis Proxies** | Anabolic agents for systemic use | A14 | <5 (0) |
| **Diagnosis Proxies** | Other alimentary tract and metabolism products | A16 | 21 (0) |
| **Diagnosis Proxies** | Antithrombotic agents | B01 | 608,307 (24.95) |
| **Diagnosis Proxies** | Antihemorrhagics | B02 | 73,671 (3.02) |
| **Diagnosis Proxies** | Antianemic preparations | B03 | 278,031 (11.4) |
| **Diagnosis Proxies** | Blood substitutes and perfusion solutions | B05 | 1,058 (0.04) |
| **Diagnosis Proxies** | Cardiac therapy | C01 | 224,151 (9.19) |
| **Diagnosis Proxies** | Antihypertensives | C02 | 35,258 (1.45) |
| **Diagnosis Proxies** | Diuretics | C03 | 604,210 (24.78) |
| **Diagnosis Proxies** | Peripheral vasodilators | C04 | 383 (0.02) |
| **Diagnosis Proxies** | Vasoprotectives | C05 | 603,499 (24.75) |
| **Diagnosis Proxies** | Beta blocking agents | C07 | 504,114 (20.68) |
| **Diagnosis Proxies** | Calcium channel blockers | C08 | 600,019 (24.61) |
| **Diagnosis Proxies** | Agents acting on the renin-angiotensin system | C09 | 836,516 (34.31) |
| **Diagnosis Proxies** | Lipid modifying agents | C10 | 781,658 (32.06) |
| **Diagnosis Proxies** | Antifungals for dermatological use | D01 | 931,014 (38.19) |
| **Diagnosis Proxies** | Emollients and protectives | D02 | 6,508 (0.27) |
| **Diagnosis Proxies** | Preparations for treatment of wounds and ulcers | D03 | 78 (0) |
| **Diagnosis Proxies** | Antipruritics, incl. antihistamines, anesthetics, etc. | D04 | 12,021 (0.49) |
| **Diagnosis Proxies** | Antipsoriatics | D05 | 88,893 (3.65) |
| **Diagnosis Proxies** | Antibiotics and chemotherapeutics for dermatological use | D06 | 643,421 (26.39) |
| **Diagnosis Proxies** | Corticosteroids, dermatological preparations | D07 | 1,253,225 (51.4) |
| **Diagnosis Proxies** | Antiseptics and disinfectants | D08 | 620 (0.03) |
| **Diagnosis Proxies** | Medicated dressings | D09 | 111 (0) |
| **Diagnosis Proxies** | Anti-acne preparations | D10 | 100,199 (4.11) |
| **Diagnosis Proxies** | Other dermatological preparations | D11 | 131,881 (5.41) |
| **Diagnosis Proxies** | Gynecological antiinfectives and antiseptics | G01 | 142,175 (5.83) |
| **Diagnosis Proxies** | Other gynecologicals | G02 | 180,722 (7.41) |
| **Diagnosis Proxies** | Sex hormones and modulators of the genital system | G03 | 617,652 (25.33) |
| **Diagnosis Proxies** | Urologicals | G04 | 439,829 (18.04) |
| **Diagnosis Proxies** | Pituitary and hypothalamic hormones and analogues | H01 | 26,378 (1.08) |
| **Diagnosis Proxies** | Corticosteroids for systemic use | H02 | 544,772 (22.34) |
| **Diagnosis Proxies** | Thyroid therapy | H03 | 158,170 (6.49) |
| **Diagnosis Proxies** | Pancreatic hormones | H04 | 7,113 (0.29) |
| **Diagnosis Proxies** | Calcium homeostasis | H05 | 483 (0.02) |
| **Diagnosis Proxies** | Antibacterials for systemic use | J01 | 2,183,851 (89.57) |
| **Diagnosis Proxies** | Antimycotics for systemic use | J02 | 383,167 (15.72) |
| **Diagnosis Proxies** | Antimycobacterials | J04 | 4,716 (0.19) |
| **Diagnosis Proxies** | Antivirals for systemic use | J05 | 309,529 (12.7) |
| **Diagnosis Proxies** | Immune sera and immunoglobulins | J06 | 1,071 (0.04) |
| **Diagnosis Proxies** | Vaccines | J07 | 225,930 (9.27) |
| **Diagnosis Proxies** | Antineoplastic agents | L01 | 12,845 (0.53) |
| **Diagnosis Proxies** | Endocrine therapy | L02 | 12,135 (0.5) |
| **Diagnosis Proxies** | Immunostimulants | L03 | 6 (0) |
| **Diagnosis Proxies** | Immunosuppressants | L04 | 58,179 (2.39) |
| **Diagnosis Proxies** | Antiinflammatory and antirheumatic products | M01 | 1,788,201 (73.34) |
| **Diagnosis Proxies** | Topical products for joint and muscular pain | M02 | 86,968 (3.57) |
| **Diagnosis Proxies** | Muscle relaxants | M03 | 262,900 (10.78) |
| **Diagnosis Proxies** | Antigout preparations | M04 | 94,271 (3.87) |
| **Diagnosis Proxies** | Drugs for treatment of bone diseases | M05 | <5 (0) |
| **Diagnosis Proxies** | Other drugs for disorders of the musculo-skeletal system | M09 | 4,248 (0.17) |
| **Diagnosis Proxies** | Anesthetics | N01 | 24,584 (1.01) |
| **Diagnosis Proxies** | Analgesics | N02 | 1,648,611 (67.62) |
| **Diagnosis Proxies** | Antiepileptics | N03 | 106,628 (4.37) |
| **Diagnosis Proxies** | Anti-parkinson drugs | N04 | 75,512 (3.1) |
| **Diagnosis Proxies** | Psycholeptics | N05 | 712,443 (29.22) |
| **Diagnosis Proxies** | Psychoanaleptics | N06 | 608,168 (24.94) |
| **Diagnosis Proxies** | Other nervous system drugs | N07 | 214,633 (8.8) |
| **Diagnosis Proxies** | Antiprotozoals | P01 | 627,321 (25.73) |
| **Diagnosis Proxies** | Anthelmintics | P02 | 169,252 (6.94) |
| **Diagnosis Proxies** | Ectoparasiticides, incl. scabicides, insecticides and repellents | P03 | 28,419 (1.17) |
| **Diagnosis Proxies** | Nasal preparations | R01 | 669,352 (27.45) |
| **Diagnosis Proxies** | Throat preparations | R02 | 46,843 (1.92) |
| **Diagnosis Proxies** | Drugs for obstructive airway diseases | R03 | 577,995 (23.71) |
| **Diagnosis Proxies** | Cough and cold preparations | R05 | 710,371 (29.14) |
| **Diagnosis Proxies** | Antihistamines for systemic use | R06 | 635,720 (26.07) |
| **Diagnosis Proxies** | Other respiratory system products | R07 | <5 (0) |
| **Diagnosis Proxies** | Ophthalmologicals | S01 | 1,371,597 (56.26) |
| **Diagnosis Proxies** | Otologicals | S02 | 275,957 (11.32) |
| **Diagnosis Proxies** | Ophthalmological and otological preparations | S03 | 247,955 (10.17) |
| **Diagnosis Proxies** | Allergens | V01 | 19,022 (0.78) |
| **Diagnosis Proxies** | All other therapeutic products | V03 | 960 (0.04) |
| **Diagnosis Proxies** | Diagnostic agents | V04 | 142 (0.01) |
| **Diagnosis Proxies** | All other non-therapeutic products | V07 | 1,609 (0.07) |
| **Diagnosis Proxies** | Contrast media | V08 | 39 (0) |
| **Diagnosis Proxies** | Diagnostic radiopharmaceuticals | V09 | <5 (0) |
| **Diagnosis Proxies** | Therapeutic radiopharmaceuticals | V10 | 13 (0) |
| **Osteoporosis Risk** | Proton pump inhibitors | A02BC | 1,041,135 (42.7) |
| **Osteoporosis Risk** | Thiazolidinediones | A10BG | 1,495 (0.06) |
| **Osteoporosis Risk** | Heparin group | B01AB | 18,800 (0.77) |
| **Osteoporosis Risk** | Vitamin k antagonists | B01AA | 81,503 (3.34) |
| **Osteoporosis Risk** | Glucocorticoids | H02AB | 544,488 (22.33) |
| **Osteoporosis Risk** | Folic acid analogues | L01BA | 147 (0.01) |
| **Osteoporosis Risk** | Nitrogen mustard analogues | L01AA | 217 (0.01) |
| **Osteoporosis Risk** | Gonadotropin releasing hormone analogues | L02AE | 9,866 (0.4) |
| **Osteoporosis Risk** | Aromatase inhibitors | L02BG | 1,022 (0.04) |
| **Osteoporosis Risk** | Calcineurin inhibitors | L04AD | 1,073 (0.04) |
| **Osteoporosis Risk** | Hydantoin derivatives | N03AB | 1,058 (0.04) |
| **Osteoporosis Risk** | Carboxamide derivatives | N03AF | 14,866 (0.61) |
| **Osteoporosis Risk** | Fatty acid derivatives | N03AG | 16,361 (0.67) |
| **Osteoporosis Risk** | Barbiturates and derivatives | N03AA | 3,622 (0.15) |
| **Osteoporosis Risk** | Selective serotonin reuptake inhibitors | N06AB | 393,585 (16.14) |
|  |  | N06AX | 145,631 (5.97) |
| **Fall Risk** | Synthetic anticholinergics, quaternary ammonium compounds | A03AB | 4,434 (0.18) |
| **Fall Risk** | Belladonna alkaloids, tertiary amines | A03BA | 1,815 (0.07) |
| **Fall Risk** | Belladonna alkaloids, semisynthetic, quaternary ammonium compounds | A03BB | 11,705 (0.48) |
| **Fall Risk** | Antiarrhythmics, class ic | C01BC | 7,569 (0.31) |
| **Fall Risk** | Antiarrhythmics, class iii | C01BD | 22,697 (0.93) |
| **Fall Risk** | Organic nitrates | C01DA | 150,584 (6.18) |
| **Fall Risk** | Other vasodilators used in cardiac diseases | C01DX | 1,947 (0.08) |
| **Fall Risk** | Digitalis glycosides | C01AA | 38,674 (1.59) |
| **Fall Risk** | Methyldopa | C02AB | 1,185 (0.05) |
| **Fall Risk** | Imidazoline receptor agonists | C02AC | 13,361 (0.55) |
| **Fall Risk** | Alpha-adrenoreceptor antagonists | C02CA | 23,594 (0.97) |
| **Fall Risk** | Thiazides and potassium in combination | C03AB | 449,375 (18.43) |
| **Fall Risk** | Sulfonamides, plain | C03BA | 9,029 (0.37) |
| **Fall Risk** | Sulfonamides, plain | C03CA | 24,3221 (9.98) |
| **Fall Risk** | Sulfonamides and potassium in combination | C03CB | 1,524 (0.06) |
| **Fall Risk** | Aldosterone antagonists | C03DA | 100,357 (4.12) |
| **Fall Risk** | Other potassium-sparing agents | C03DB | 61 (0) |
| **Fall Risk** | Low-ceiling diuretics and potassium-sparing agents | C03EA | 24,637 (1.01) |
| **Fall Risk** | High-ceiling diuretics and potassium-sparing agents | C03EB | 2,294 (0.09) |
| **Fall Risk** | Vasopressin antagonists | C03XA | 7 (0) |
| **Fall Risk** | Thiazides, plain | C03AA | 36,060 (1.48) |
| **Fall Risk** | Imidazoline derivatives | C04AB | <5 (0) |
| **Fall Risk** | Nicotinic acid and derivatives | C04AC | 7 (0) |
| **Fall Risk** | Purine derivatives | C04AD | 376 (0.02) |
| **Fall Risk** | Beta blocking agents, selective | C07AB | 421,900 (17.3) |
| **Fall Risk** | Alpha and beta blocking agents | C07AG | 47,829 (1.96) |
| **Fall Risk** | Beta blocking agents, selective, and thiazides | C07BB | 2,120 (0.09) |
| **Fall Risk** | Beta blocking agents, selective, and other diuretics | C07CB | 2,464 (0.1) |
| **Fall Risk** | Beta blocking agents and calcium channel blockers | C07FB | 606 (0.02) |
| **Fall Risk** | Beta blocking agents, non-selective | C07AA | 75,931 (3.11) |
| **Fall Risk** | Dihydropyridine derivatives | C08CA | 580,395 (23.8) |
| **Fall Risk** | Phenylalkylamine derivatives | C08DA | 30,337 (1.24) |
| **Fall Risk** | Benzothiazepine derivatives | C08DB | 8,055 (0.33) |
| **Fall Risk** | Ace inhibitors and diuretics | C09BA | 14,8647 (6.1) |
| **Fall Risk** | Ace inhibitors and calcium channel blockers | C09BB | 217 (0.01) |
| **Fall Risk** | Angiotensin ii receptor blockers (arbs), plain | C09CA | 483,946 (19.85) |
| **Fall Risk** | Angiotensin ii receptor blockers (arbs) and diuretics | C09DA | 207,636 (8.52) |
| **Fall Risk** | Angiotensin ii receptor blockers (arbs) and calcium channel blockers | C09DB | 5,369 (0.22) |
| **Fall Risk** | Angiotensin ii receptor blockers (arbs), other combinations | C09DX | 4,751 (0.19) |
| **Fall Risk** | Renin-inhibitors | C09XA | 3,913 (0.16) |
| **Fall Risk** | Ace inhibitors, plain | C09AA | 503,251 (20.64) |
| **Fall Risk** | Drugs for urinary frequency and incontinence | G04BD | 95,244 (3.91) |
| **Fall Risk** | Alpha-adrenoreceptor antagonists | G04CA | 159,060 (6.52) |
| **Fall Risk** | Other centrally acting agents | M03BX | 38,520 (1.58) |
| **Fall Risk** | Hydantoin derivatives | N03AB | 1,058 (0.04) |
| **Fall Risk** | Succinimide derivatives | N03AD | 73 (0) |
| **Fall Risk** | Benzodiazepine derivatives | N03AE | 14,368 (0.59) |
| **Fall Risk** | Carboxamide derivatives | N03AF | 21,453 (0.88) |
| **Fall Risk** | Fatty acid derivatives | N03AG | 16,459 (0.68) |
| **Fall Risk** | Other antiepileptics | N03AX | 69,360 (2.84) |
| **Fall Risk** | Barbiturates and derivatives | N03AA | 6,650 (0.27) |
| **Fall Risk** | Ethers chemically close to antihistamines | N04AB | 7,979 (0.33) |
| **Fall Risk** | Tertiary amines | N04AA | 4,743 (0.19) |
| **Fall Risk** | Phenothiazines with piperazine structure | N05AB | 4,514 (0.19) |
| **Fall Risk** | Phenothiazines with piperidine structure | N05AC | 393 (0.02) |
| **Fall Risk** | Butyrophenone derivatives | N05AD | 6,706 (0.28) |
| **Fall Risk** | Indole derivatives | N05AE | 4,023 (0.17) |
| **Fall Risk** | Thioxanthene derivatives | N05AF | 62,138 (2.55) |
| **Fall Risk** | Diphenylbutylpiperidine derivatives | N05AG | 1,359 (0.06) |
| **Fall Risk** | Diazepines, oxazepines, thiazepines and oxepines | N05AH | 103,788 (4.26) |
| **Fall Risk** | Benzamides | N05AL | 1,928 (0.08) |
| **Fall Risk** | Lithium | N05AN | 12,657 (0.52) |
| **Fall Risk** | Other antipsychotics | N05AX | 37,612 (1.54) |
| **Fall Risk** | Benzodiazepine derivatives | N05BA | 325,917 (13.37) |
| **Fall Risk** | Diphenylmethane derivatives | N05BB | 27,632 (1.13) |
| **Fall Risk** | Aldehydes and derivatives | N05CC | <5 (0) |
| **Fall Risk** | Benzodiazepine derivatives | N05CD | 44,301 (1.82) |
| **Fall Risk** | Benzodiazepine related drugs | N05CF | 408,255 (16.74) |
| **Fall Risk** | Melatonin receptor agonists | N05CH | 165,565 (6.79) |
| **Fall Risk** | Phenothiazines with aliphatic side-chain | N05AA | 15,591 (0.64) |
| **Fall Risk** | Selective serotonin reuptake inhibitors | N06AB | 393,585 (16.14) |
| **Fall Risk** | Monoamine oxidase inhibitors, non-selective | N06AF | 858 (0.04) |
| **Fall Risk** | Monoamine oxidase a inhibitors | N06AG | 526 (0.02) |
| **Fall Risk** | Other antidepressants | N06AX | 321,647 (13.19) |
| **Fall Risk** | Non-selective monoamine reuptake inhibitors | N06AA | 147,488 (6.05) |
| **Fall Risk** | Antivertigo preparations | N07CA | 11,454 (0.47) |
| **Fall Risk** | Phenothiazine derivatives | R06AD | 88,400 (3.63) |
| **Fall Risk** | Piperazine derivatives | R06AE | 24,963 (1.02) |
| **Fall Risk** | Aminoalkyl ethers | R06AA | 17,582 (0.72) |
| **Fall Risk (6 months f.I.)** | Synthetic anticholinergics, quaternary ammonium compounds | A03AB | <5 (0) |
| **Fall Risk (6 months f.I.)** | Belladonna alkaloids, tertiary amines | A03BA | 371 (0.02) |
| **Fall Risk (6 months f.I.)** | Belladonna alkaloids, semisynthetic, quaternary ammonium compounds | A03BB | 108 (0) |
| **Fall Risk (6 months f.I.)** | Antiarrhythmics, class ic | C01BC | 2,142 (0.09) |
| **Fall Risk (6 months f.I.)** | Antiarrhythmics, class iii | C01BD | 5,508 (0.23) |
| **Fall Risk (6 months f.I.)** | Organic nitrates | C01DA | 31,661 (1.3) |
| **Fall Risk (6 months f.I.)** | Other vasodilators used in cardiac diseases | C01DX | 710 (0.03) |
| **Fall Risk (6 months f.I.)** | Digitalis glycosides | C01AA | 19,548 (0.8) |
| **Fall Risk (6 months f.I.)** | Imidazoline receptor agonists | C02AC | 5,595 (0.23) |
| **Fall Risk (6 months f.I.)** | Alpha-adrenoreceptor antagonists | C02CA | 10,047 (0.41) |
| **Fall Risk (6 months f.I.)** | Thiazides and potassium in combination | C03AB | 147,956 (6.07) |
| **Fall Risk (6 months f.I.)** | Sulfonamides, plain | C03BA | 3,340 (0.14) |
| **Fall Risk (6 months f.I.)** | Sulfonamides, plain | C03CA | 108,445 (4.45) |
| **Fall Risk (6 months f.I.)** | Aldosterone antagonists | C03DA | 54,191 (2.22) |
| **Fall Risk (6 months f.I.)** | Other potassium-sparing agents | C03DB | 40 (0) |
| **Fall Risk (6 months f.I.)** | Low-ceiling diuretics and potassium-sparing agents | C03EA | 758 (0.03) |
| **Fall Risk (6 months f.I.)** | High-ceiling diuretics and potassium-sparing agents | C03EB | 854 (0.04) |
| **Fall Risk (6 months f.I.)** | Vasopressin antagonists | C03XA | <5 (0) |
| **Fall Risk (6 months f.I.)** | Thiazides, plain | C03AA | 10,279 (0.42) |
| **Fall Risk (6 months f.I.)** | Nicotinic acid and derivatives | C04AC | <5 (0) |
| **Fall Risk (6 months f.I.)** | Purine derivatives | C04AD | 14 (0) |
| **Fall Risk (6 months f.I.)** | Beta blocking agents, selective | C07AB | 247,071 (10.13) |
| **Fall Risk (6 months f.I.)** | Alpha and beta blocking agents | C07AG | 27,557 (1.13) |
| **Fall Risk (6 months f.I.)** | Beta blocking agents, selective, and thiazides | C07BB | 596 (0.02) |
| **Fall Risk (6 months f.I.)** | Beta blocking agents, selective, and other diuretics | C07CB | 195 (0.01) |
| **Fall Risk (6 months f.I.)** | Beta blocking agents, non-selective | C07AA | 15,759 (0.65) |
| **Fall Risk (6 months f.I.)** | Dihydropyridine derivatives | C08CA | 354,628 (14.55) |
| **Fall Risk (6 months f.I.)** | Phenylalkylamine derivatives | C08DA | 9,758 (0.4) |
| **Fall Risk (6 months f.I.)** | Benzothiazepine derivatives | C08DB | 2,425 (0.1) |
| **Fall Risk (6 months f.I.)** | Ace inhibitors and diuretics | C09BA | 39,414 (1.62) |
| **Fall Risk (6 months f.I.)** | Ace inhibitors and calcium channel blockers | C09BB | <5 (0) |
| **Fall Risk (6 months f.I.)** | Angiotensin ii receptor blockers (arbs), plain | C09CA | 320,534 (13.15) |
| **Fall Risk (6 months f.I.)** | Angiotensin ii receptor blockers (arbs) and diuretics | C09DA | 77,794 (3.19) |
| **Fall Risk (6 months f.I.)** | Angiotensin ii receptor blockers (arbs) and calcium channel blockers | C09DB | 82 (0) |
| **Fall Risk (6 months f.I.)** | Angiotensin ii receptor blockers (arbs), other combinations | C09DX | 3,567 (0.15) |
| **Fall Risk (6 months f.I.)** | Renin-inhibitors | C09XA | 222 (0.01) |
| **Fall Risk (6 months f.I.)** | Ace inhibitors, plain | C09AA | 219,425 (9) |
| **Fall Risk (6 months f.I.)** | Drugs for urinary frequency and incontinence | G04BD | 29,471 (1.21) |
| **Fall Risk (6 months f.I.)** | Alpha-adrenoreceptor antagonists | G04CA | 60,325 (2.47) |
| **Fall Risk (6 months f.I.)** | Other centrally acting agents | M03BX | 11,096 (0.46) |
| **Fall Risk (6 months f.I.)** | Hydantoin derivatives | N03AB | 159 (0.01) |
| **Fall Risk (6 months f.I.)** | Succinimide derivatives | N03AD | 28 (0) |
| **Fall Risk (6 months f.I.)** | Benzodiazepine derivatives | N03AE | 3,912 (0.16) |
| **Fall Risk (6 months f.I.)** | Carboxamide derivatives | N03AF | 6,866 (0.28) |
| **Fall Risk (6 months f.I.)** | Fatty acid derivatives | N03AG | 6,543 (0.27) |
| **Fall Risk (6 months f.I.)** | Other antiepileptics | N03AX | 30,652 (1.26) |
| **Fall Risk (6 months f.I.)** | Barbiturates and derivatives | N03AA | 1,697 (0.07) |
| **Fall Risk (6 months f.I.)** | Ethers chemically close to antihistamines | N04AB | 1,766 (0.07) |
| **Fall Risk (6 months f.I.)** | Tertiary amines | N04AA | 1,355 (0.06) |
| **Fall Risk (6 months f.I.)** | Phenothiazines with piperazine structure | N05AB | 294 (0.01) |
| **Fall Risk (6 months f.I.)** | Butyrophenone derivatives | N05AD | 1,328 (0.05) |
| **Fall Risk (6 months f.I.)** | Indole derivatives | N05AE | 790 (0.03) |
| **Fall Risk (6 months f.I.)** | Thioxanthene derivatives | N05AF | 8,913 (0.37) |
| **Fall Risk (6 months f.I.)** | Diphenylbutylpiperidine derivatives | N05AG | 259 (0.01) |
| **Fall Risk (6 months f.I.)** | Diazepines, oxazepines, thiazepines and oxepines | N05AH | 40,279 (1.65) |
| **Fall Risk (6 months f.I.)** | Benzamides | N05AL | 394 (0.02) |
| **Fall Risk (6 months f.I.)** | Lithium | N05AN | 5,117 (0.21) |
| **Fall Risk (6 months f.I.)** | Other antipsychotics | N05AX | 11,838 (0.49) |
| **Fall Risk (6 months f.I.)** | Benzodiazepine derivatives | N05BA | 42,339 (1.74) |
| **Fall Risk (6 months f.I.)** | Diphenylmethane derivatives | N05BB | 3,250 (0.13) |
| **Fall Risk (6 months f.I.)** | Benzodiazepine derivatives | N05CD | 3,915 (0.16) |
| **Fall Risk (6 months f.I.)** | Benzodiazepine related drugs | N05CF | 66,191 (2.71) |
| **Fall Risk (6 months f.I.)** | Melatonin receptor agonists | N05CH | 29,961 (1.23) |
| **Fall Risk (6 months f.I.)** | Selective serotonin reuptake inhibitors | N06AB | 121,950 (5) |
| **Fall Risk (6 months f.I.)** | Monoamine oxidase inhibitors, non-selective | N06AF | 245 (0.01) |
| **Fall Risk (6 months f.I.)** | Other antidepressants | N06AX | 100,126 (4.11) |
| **Fall Risk (6 months f.I.)** | Non-selective monoamine reuptake inhibitors | N06AA | 26,854 (1.1) |
| **Fall Risk (6 months f.I.)** | Antivertigo preparations | N07CA | 344 (0.01) |
| **Fall Risk (6 months f.I.)** | Phenothiazine derivatives | R06AD | 16,551 (0.68) |
| **Fall Risk (6 months f.I.)** | Piperazine derivatives | R06AE | 1,112 (0.05) |
| **Fall Risk (6 months f.I.)** | Aminoalkyl ethers | R06AA | 1,329 (0.05) |

Abbreviations: 6 months f.I. – last redemption < 6 months from index.

**Supplementary Table 7. Comparison of Training and Testing Sample**

| Cohort | Training | Validation | Testing |
| --- | --- | --- | --- |
| Sample Size (%) | 1,462,650 (60.0) | 487,700 (20.0) | 487,790 (20.0) |
| Median Age (IQR) | 61.4 (18.5) | 61.4 (18.5) | 61.4 (18.5) |
| Female (%) | 718,849 (49.1) | 239,706.0 (49.2) | 239,661 (49.1) |
| Death in 2022 (%) | 25,768 (1.8) | 8,438 (1.7) | 8,465 (1.7) |
| Emigration in 2022 (%) | 2,028 (0.1) | 697 (0.1) | 677 (0.1) |
| MOF in 2022 (%) | 13,343 (0.9) | 4,312 (0.9) | 4,387 (0.9) |
| HF in 2022 (%) | 3,578 (0.2) | 1,135 (0.2) | 1,165 (0.2) |

The dataset splits follow Möller et al. (2025). Due to the removal of individuals with incomplete lookback in this study, the final sample size deviates slightly from the exact split, though without any impact on model training.

**Supplementary Table 8. Hyperparameter Search Space and Selections**

Hyperparameter search was carried out using Bayesian optimization by the mlrMBO R package, with a binary classification objective using the metric *AUC* with deactivated feature pre-filtering and early stopping after 200 iterations of no improvement in the training dataset (0.6). In a first round, the optimal learning rate was determined. Next, num_leaves, min_data_in_leaf, feature_fraction, and bagging_fraction, and max_depth hyperparameters were selected. Information on hyperparameters for MOF and for HF outcomes is shown below

|  |  | Major Osteoporotic Fractures | | | Hip Fractures |
| --- | --- | --- | --- | --- | --- |
| Hyperparameter | Search Space | Female | Male | All | All |
| learning_rate | 0.01:0.3 | 0.01 | 0.0663 | 0.0489 | 0.0479 |
| Num_leaves | 4:60 | 60 | 9 | 20 | 30 |
| Min_data_in_leaf | 500:4000 | 500 | 2880 | 2752 | 3397 |
| feature_fraction | 0.2:1 | 0.768 | 0.824 | 0.691 | 0.314 |
| bagging_fraction | 0.5:1 | 0.842 | 0.5 | 0.5 | 0.545 |
| max_depth | -1:12, Iteratively | -1 | 11 | 5 | 3 |
| Is_unbalance | Iteratively | TRUE | TRUE | TRUE | TRUE |
| Objective | Task directed | Binary | | | |
| Metric | Task directed | AUC | | | |
| Boosting | DART, GBDT | DART | | | |
| Num_iterations | Iteratively | 2000 | | | |
| Early stopping | Task directed | 200 | | | |
| Feature_pre_filter | Task directed | FALSE | | | |
| Force_row_wise | Task directed | TRUE | | | |
| Deterministic | Task directed | TRUE | | | |

**Supplementary Table 9. Model Analytics by Probability Thresholds for Major Osteoporotic Fractures in the Testing Sample**

| Cutoff | Acc (%) | PPV | NPV | k | Sens (%) | Spec (%) | F1 | Balanced Acc (%) | TP | TN | FP | FN | N  High Risk_2022_ | N DXA_2022_ | N Osteo _2022_ | N MOF | N MOF  No DXA | N Correct High Risk | N No MOF | Mean Age  High Risk |
| --- | --- | --- | --- | --- | --- | --- | --- | --- | --- | --- | --- | --- | --- | --- | --- | --- | --- | --- | --- | --- |
| 0.00 | 0.90 | 0.009 | NaN | 0.000 | 100.00 | 0.00 | 0.018 | 50.00 | 4,387 | 0 | 483,403 | 0 | 248,129 | 3,187 | 1,326 | 1,385 | 1,196 | 703 | 246,744 | 62.99 |
| 0.05 | 0.90 | 0.009 | 1 | 0.000 | 100.00 | 0.00 | 0.018 | 50.00 | 4,387 | 9 | 483,394 | 0 | 248,126 | 3,187 | 1,326 | 1,385 | 1,196 | 703 | 246,741 | 62.99 |
| 0.10 | 1.65 | 0.009 | 0.999 | 0.000 | 99.91 | 0.76 | 0.018 | 50.34 | 4,383 | 3,682 | 479,721 | 4 | 247,395 | 3,182 | 1,325 | 1,383 | 1,194 | 703 | 246,012 | 63.03 |
| 0.15 | 8.86 | 0.01 | 0.998 | 0.001 | 98.47 | 8.05 | 0.019 | 53.26 | 4,320 | 38,914 | 444,489 | 67 | 226,960 | 3,138 | 1,319 | 1,346 | 1,158 | 703 | 225,614 | 64.26 |
| 0.20 | 22.07 | 0.011 | 0.998 | 0.004 | 95.40 | 21.41 | 0.022 | 58.40 | 4,185 | 103,485 | 379,918 | 202 | 174,450 | 2,911 | 1,270 | 1,237 | 1,054 | 703 | 173,213 | 67.38 |
| 0.25 | 34.26 | 0.013 | 0.998 | 0.007 | 92.45 | 33.73 | 0.025 | 63.09 | 4,056 | 163,075 | 320,328 | 331 | 126,797 | 2,521 | 1,194 | 1,135 | 964 | 703 | 125,662 | 70.24 |
| 0.30 | 44.66 | 0.014 | 0.998 | 0.010 | 88.51 | 44.26 | 0.028 | 66.39 | 3,883 | 213,947 | 269,456 | 504 | 93,128 | 2,137 | 1,078 | 1,036 | 883 | 703 | 92,092 | 72.38 |
| 0.35 | 52.29 | 0.016 | 0.997 | 0.013 | 84.59 | 52.00 | 0.031 | 68.29 | 3,711 | 251,356 | 232,047 | 676 | 69,408 | 1,752 | 921 | 933 | 795 | 703 | 68,475 | 73.95 |
| 0.3864910 | 57.28 | 0.017 | 0.997 | 0.016 | 81.13 | 57.06 | 0.033 | 69.09 | 3,559 | 275,827 | 207,576 | 828 | 56,869 | 1,524 | 840 | 864 | 735 | 703 | 56,005 | 74.87 |
| 0.40 | 59.30 | 0.017 | 0.997 | 0.017 | 79.74 | 59.12 | 0.034 | 69.43 | 3,498 | 285,769 | 197,634 | 889 | 52,894 | 1,433 | 808 | 841 | 718 | 703 | 52,053 | 75.16 |
| 0.45 | 66.39 | 0.02 | 0.996 | 0.021 | 74.06 | 66.32 | 0.038 | 70.19 | 3,249 | 320,610 | 162,793 | 1,138 | 40,544 | 1,159 | 697 | 770 | 656 | 703 | 39,774 | 76.17 |
| 0.4872454 | 71.08 | 0.021 | 0.996 | 0.024 | 69.18 | 71.10 | 0.041 | 70.14 | 3,035 | 343,694 | 139,709 | 1,352 | 33,137 | 968 | 610 | 703 | 599 | 703 | 32,434 | 76.93 |
| 0.50 | 72.63 | 0.022 | 0.996 | 0.025 | 67.34 | 72.67 | 0.042 | 70.00 | 2,954 | 351,310 | 132,093 | 1,433 | 30,913 | 921 | 585 | 678 | 578 | 678 | 30,235 | 77.17 |
| 0.55 | 78.65 | 0.025 | 0.995 | 0.031 | 59.68 | 78.82 | 0.048 | 69.25 | 2,618 | 381,023 | 102,380 | 1,769 | 22,975 | 716 | 471 | 572 | 496 | 572 | 22,403 | 78.07260 |
| 0.5862841 | 82.60 | 0.028 | 0.995 | 0.036 | 53.41 | 82.86 | 0.052 | 68.13 | 2,343 | 400,549 | 82,854 | 2,044 | 18,266 | 565 | 400 | 504 | 439 | 504 | 17,762 | 78.73 |
| 0.60 | 84.01 | 0.029 | 0.995 | 0.038 | 50.90 | 84.31 | 0.054 | 67.61 | 2,233 | 407,579 | 75,824 | 2,154 | 16,676 | 502 | 369 | 476 | 413 | 476 | 16,200 | 78.94 |
| 0.65 | 88.28 | 0.034 | 0.994 | 0.046 | 43.36 | 88.69 | 0.062 | 66.02 | 1,902 | 428,726 | 54,677 | 2,485 | 11,528 | 343 | 265 | 391 | 343 | 391 | 11,137 | 79.60 |
| 0.70 | 91.64 | 0.038 | 0.994 | 0.054 | 34.49 | 92.16 | 0.069 | 63.33 | 1,513 | 445,519 | 37,884 | 2,874 | 7,390 | 208 | 169 | 283 | 247 | 283 | 7,107 | 79.87 |
| 0.75 | 94.38 | 0.045 | 0.993 | 0.062 | 25.96 | 95.00 | 0.077 | 60.48 | 1,139 | 459,218 | 24,185 | 3,248 | 4,376 | 120 | 109 | 198 | 175 | 198 | 4,178 | 79.86 |
| 0.80 | 96.52 | 0.052 | 0.992 | 0.067 | 16.82 | 97.24 | 0.080 | 57.03 | 738 | 470,067 | 13,336 | 3,649 | 2,207 | 68 | 70 | 115 | 102 | 115 | 2,092 | 79.15 |
| 0.85 | 98.06 | 0.061 | 0.992 | 0.060 | 8.02 | 98.88 | 0.069 | 53.45 | 352 | 477,979 | 5,424 | 4,035 | 903 | 28 | 34 | 49 | 39 | 49 | 854 | 77.87 |
| 0.90 | 98.83 | 0.069 | 0.991 | 0.031 | 2.39 | 99.71 | 0.036 | 51.05 | 105 | 481,994 | 1,409 | 4,282 | 238 | 6 | 7 | 19 | Censored. | 19 | 219 | 75.30 |
| 0.95 | 99.09 | 0.13 | 0.991 | 0.004 | 0.21 | 99.99 | 0.004 | 50.10 | 9 | 483,343 | 60 | 4,378 | 12 | 0 | 0 | 0 | 0 | 0 | 12 | 69.00 |
| 1.00 | 99.10 | NaN | 0.991 | 0.000 | 0.00 | 100.00 |  | 50.00 | 0 | 483,403 | 0 | 4,387 | 0 | 0 | 0 | 0 | 0 | 0 | 0 | - |

Youden’s Index is shaded in grey, and female and male specific Youden’s is shaded in red and blue, respectively. Abbreviations: Acc – Accuracy; NPV – negative predictive value; PPV – positive predictive value; Sens – sensitivity; Spec – specificity; TP – true positives; FP – false positives; FN – false negatives; TP – true positives; DXA – dual x-ray absorptiometry; Osteo – Primary osteoporosis diagnosis or medication; NA – Not applicable.

**Supplementary Table 10. Features included in the Model for Major Osteoporotic Fractures and corresponding mean |SHAP| values**

| Rank | \|SHAP\| | Type | Code | Definition |
| --- | --- | --- | --- | --- |
| 1 | 0.49295 | ATC Diagnosis Proxies |  | Age |
| 2 | 0.35150 | ATC Diagnosis Proxies |  | Sex |
| 3 | 0.07145 | Comorbidities |  | N Diagnoses within 1 Year f.I. |
| 4 | 0.06393 | ICD-10 Diagnosis | S52 | Fracture of forearm |
| 5 | 0.04574 | ICD-10 Diagnosis | S42 | Fracture of shoulder and upper arm |
| 6 | 0.02898 | ICD-10 Diagnosis | S62 | Fracture at wrist and hand level |
| 7 | 0.02674 | Comorbidities |  | N Diagnoses within 2-5 Years f.I. |
| 8 | 0.02558 | ATC Diagnosis Proxies | C05 | Vasoprotectives |
| 9 | 0.02401 | ATC Diagnosis Proxies | N07 | Other nervous system drugs |
| 10 | 0.02099 | ATC Fall Risk | C03CA | Sulfonamides, plain |
| 11 | 0.02035 | ATC Fall Risk | N06AB | Selective serotonin reuptake inhibitors |
| 12 | 0.02020 | ICD-10 Diagnosis | S82 | Fracture of lower leg, including ankle |
| 13 | 0.01926 | ATC Fall Risk | C03AB | Thiazides and potassium in combination |
| 14 | 0.01922 | ATC Diagnosis Proxies | A06 | Drugs for constipation |
| 15 | 0.01864 | Comorbidities |  | Charlson's Comorbidity Index |
| 16 | 0.01656 | ICD-10 Risk Factors |  | Alcohol Use |
| 17 | 0.01557 | ICD-10 Diagnosis | S01 | Open wound of head |
| 18 | 0.01545 | ATC Diagnosis Proxies | J01 | Antibacterials for systemic use |
| 19 | 0.01469 | ATC Osteoporosis Risk | A02BC | Proton pump inhibitors |
| 20 | 0.01450 | ATC Fall Risk | C09DA | Angiotensin ii receptor blockers (arbs) and diuretics |
| 21 | 0.01402 | ATC Diagnosis Proxies | B03 | Antianemic preparations |
| 22 | 0.01399 | ICD-10 Diagnosis | S93 | Dislocation, sprain and strain of joints and ligaments at ankle andÂ foot level |
| 23 | 0.01143 | ICD-10 Diagnosis | S63 | Dislocation and sprain of joints and ligaments at wrist / hand level |
| 24 | 0.01110 | ATC Fall Risk | C09CA | Angiotensin ii receptor blockers (arbs), plain |
| 25 | 0.01099 | ICD-10 Diagnosis | S92 | Fracture of foot and toe, except ankle |
| 26 | 0.01008 | Polypharmacy |  | N Presciptions Therapeutic Subgroup |
| 27 | 0.00974 | ATC Fall Risk | N06AX | Other antidepressants |
| 28 | 0.00971 | ICD-10 Diagnosis | M16 | Osteoarthritis of hip |
| 29 | 0.00925 | ICD-10 Diagnosis | S72 | Fracture of femur |
| 30 | 0.00917 | ATC Diagnosis Proxies | N02 | Analgesics |
| 31 | 0.00901 | ICD-10 Diagnosis | S22 | Fracture of rib(s), sternum and thoracic spine |
| 32 | 0.00892 | ICD-10 Diagnosis | S32 | Fracture of lumbar spine and pelvis |
| 33 | 0.00876 | ATC Fall Risk | C08CA | Dihydropyridine derivatives |
| 34 | 0.00832 | ICD-10 Diagnosis | H25 | Age-related cataract |
| 35 | 0.00814 | ATC Fall Risk | C07AB | Beta blocking agents, selective |
| 36 | 0.00801 | ATC Fall Risk | C09AA | Ace inhibitors, plain |
| 37 | 0.00796 | ICD-10 Diagnosis | I63 | Cerebral infarction |
| 38 | 0.00775 | ICD-10 Diagnosis | D12 | Benign neoplasm of colon, rectum, anus and anal canal |
| 39 | 0.00744 | ATC Fall Risk | N05CF | Benzodiazepine related drugs |
| 40 | 0.00736 | ATC Osteoporosis Risk | H02AB | Glucocorticoids |
| 41 | 0.00688 | ICD-10 Diagnosis | I20 | Angina pectoris |
| 42 | 0.00678 | ATC Diagnosis Proxies | G02 | Other gynecologicals |
| 43 | 0.00676 | ATC Diagnosis Proxies | N06 | Psychoanaleptics |
| 44 | 0.00675 | ICD-10 Diagnosis | S60 | Superficial injury of wrist, hand and fingers |
| 45 | 0.00674 | ATC Diagnosis Proxies | B01 | Antithrombotic agents |
| 46 | 0.00649 | ICD-10 Diagnosis | E66 | Overweight and obesity |
| 47 | 0.00647 | ICD-10 Risk Factors |  | Risk Factor: Dementia |
| 48 | 0.00636 | ICD-10 Diagnosis | S80 | Superficial injury of knee and lower leg |
| 49 | 0.00631 | ATC Diagnosis Proxies | G04 | Urologicals |
| 50 | 0.00620 | Comorbidities |  | N Diagnoses within 6-15 Years f.I. |
| 51 | 0.00584 | ATC Fall Risk | C03DA | Aldosterone antagonists |
| 52 | 0.00583 | ATC Fall Risk | N05BA | Benzodiazepine derivatives |
| 53 | 0.00579 | ICD-10 Diagnosis | S90 | Superficial injury of ankle, foot and toes |
| 54 | 0.00552 | ICD-10 Diagnosis | S00 | Superficial injury of head |
| 55 | 0.00547 | ICD-10 Diagnosis | M85 | Other disorders of bone density and structure |
| 56 | 0.00544 | ICD-10 Diagnosis | E78 | Disorders of lipoprotein metabolism and other lipidemias |
| 57 | 0.00522 | ATC Osteoporosis Risk | N06AB | Selective serotonin reuptake inhibitors |
| 58 | 0.00509 | ICD-10 Diagnosis | N40 | Benign prostatic hyperplasia |
| 59 | 0.00507 | ICD-10 Diagnosis | K40 | Inguinal hernia |
| 60 | 0.00501 | ICD-10 Diagnosis | N92 | Excessive, frequent and irregular menstruation |
| 61 | 0.00497 | ICD-10 Diagnosis | M50 | Cervical disc disorders |
| 62 | 0.00493 | ICD-10 Diagnosis | M20 | Acquired deformities of fingers and toes |
| 63 | 0.00491 | ICD-10 Diagnosis | E87 | Other disorders of fluid, electrolyte and acid-base balance |
| 64 | 0.00476 | ICD-10 Diagnosis | R05 | Cough |
| 65 | 0.00471 | ICD-10 Diagnosis | M75 | Shoulder lesions |
| 66 | 0.00469 | ICD-10 Diagnosis | G40 | Epilepsy and recurrent seizures |
| 67 | 0.00464 | ATC Fall Risk | C09BA | Ace inhibitors and diuretics |
| 68 | 0.00460 | ATC Fall Risk | R06AD | Phenothiazine derivatives |
| 69 | 0.00450 | ATC Diagnosis Proxies | D06 | Antibiotics and chemotherapeutics for dermatological use |
| 70 | 0.00438 | ICD-10 Diagnosis | E10 | Type 1 diabetes mellitus |
| 71 | 0.00431 | ICD-10 Diagnosis | R10 | Abdominal and pelvic pain |
| 72 | 0.00415 | ATC Fall Risk | G04CA | Alpha-adrenoreceptor antagonists |
| 73 | 0.00411 | ICD-10 Diagnosis | S86 | Injury of muscle, fascia and tendon at lower leg level |
| 74 | 0.00409 | ICD-10 Diagnosis | H91 | Other and unspecified hearing loss |
| 75 | 0.00395 | ATC Fall Risk | C01AA | Digitalis glycosides |
| 76 | 0.00381 | ATC Diagnosis Proxies | N05 | Psycholeptics |
| 77 | 0.00377 | ATC Diagnosis Proxies | J02 | Antimycotics for systemic use |
| 78 | 0.00366 | ICD-10 Diagnosis | K80 | Cholelithiasis |
| 79 | 0.00350 | ATC Diagnosis Proxies | N04 | Anti-parkinson drugs |
| 80 | 0.00350 | ICD-10 Diagnosis | R42 | Dizziness and giddiness |
| 81 | 0.00340 | ICD-10 Diagnosis | J44 | Other chronic obstructive pulmonary disease |
| 82 | 0.00319 | ATC Fall Risk | N06AA | Non-selective monoamine reuptake inhibitors |
| 83 | 0.00312 | ICD-10 Diagnosis | S20 | Superficial injury of thorax |
| 84 | 0.00309 | ICD-10 Diagnosis | G47 | Sleep disorders |
| 85 | 0.00300 | ICD-10 Diagnosis | S12 | Fracture of cervical vertebra and other parts of , not elsewhere classifiedk |
| 86 | 0.00298 | ICD-10 Diagnosis | O80 | Encounter for full-term uncomplicated delivery |
| 87 | 0.00293 | ATC Diagnosis Proxies | C10 | Lipid modifying agents |
| 88 | 0.00286 | ICD-10 Diagnosis | S06 | Intracranial injury |
| 89 | 0.00286 | ATC Diagnosis Proxies | D01 | Antifungals for dermatological use |
| 90 | 0.00282 | ATC Diagnosis Proxies | S01 | Ophthalmologicals |
| 91 | 0.00276 | ICD-10 Diagnosis | D64 | Other anemias |
| 92 | 0.00270 | ICD-10 Diagnosis | R29 | Other symptoms and signs involving the nervous and musculoskeletal systems |
| 93 | 0.00261 | ICD-10 Diagnosis | T14 | Injury of unspecified body region |
| 94 | 0.00257 | ICD-10 Risk Factors |  | Alcohol Dependency |
| 95 | 0.00254 | ATC Diagnosis Proxies | S03 | Ophthalmological and otological preparations |
| 96 | 0.00254 | ICD-10 Diagnosis | R00 | Abnormalities of heart beat |
| 97 | 0.00250 | ATC Diagnosis Proxies | H03 | Thyroid therapy |
| 98 | 0.00247 | ATC Diagnosis Proxies | M01 | Antiinflammatory and antirheumatic products |
| 99 | 0.00239 | ICD-10 Diagnosis | G20 | Parkinson's disease |
| 100 | 0.00239 | ATC Fall Risk | G04BD | Drugs for urinary frequency and incontinence |
| 101 | 0.00238 | ICD-10 Diagnosis | R06 | Abnormalities of breathing |
| 102 | 0.00237 | ICD-10 Diagnosis | S30 | Superficial injury abdomen, low back, pelvis and external genitals |
| 103 | 0.00230 | ICD-10 Diagnosis | I73 | Other peripheral vascular diseases |
| 104 | 0.00228 | ICD-10 Diagnosis | F41 | Other anxiety disorders |
| 105 | 0.00228 | ICD-10 Diagnosis | N64 | Other disorders of breast |
| 106 | 0.00225 | ATC Diagnosis Proxies | V01 | Allergens |
| 107 | 0.00224 | ATC Diagnosis Proxies | D11 | Other dermatological preparations |
| 108 | 0.00222 | ICD-10 Diagnosis | M67 | Other disorders of synovium and tendon |
| 109 | 0.00217 | ATC Fall Risk | N05CH | Melatonin receptor agonists |
| 110 | 0.00213 | ICD-10 Diagnosis | A63 | Oth predominantly sexually transmitted diseases, not elsewhere classified |
| 111 | 0.00213 | ICD-10 Diagnosis | R53 | Malaise and fatigue |
| 112 | 0.00209 | ATC Diagnosis Proxies | A10 | Drugs used in diabetes |
| 113 | 0.00202 | ATC Diagnosis Proxies | N03 | Antiepileptics |
| 114 | 0.00199 | ICD-10 Diagnosis | R07 | Pain in throat and chest |
| 115 | 0.00198 | ATC Fall Risk | N05AH | Diazepines, oxazepines, thiazepines and oxepines |
| 116 | 0.00196 | ATC Fall Risk | C01DA | Organic nitrates |
| 117 | 0.00193 | ICD-10 Diagnosis | N48 | Other disorders of penis |
| 118 | 0.00191 | ATC Diagnosis Proxies | C07 | Beta blocking agents |
| 119 | 0.00191 | ATC Diagnosis Proxies | P01 | Antiprotozoals |
| 120 | 0.00184 | ATC Diagnosis Proxies | R01 | Nasal preparations |
| 121 | 0.00183 | ATC Diagnosis Proxies | R03 | Drugs for obstructive airway diseases |
| 122 | 0.00183 | ATC Fall Risk | R06AE | Piperazine derivatives |
| 123 | 0.00181 | ICD-10 Diagnosis | M65 | Synovitis and tenosynovitis |
| 124 | 0.00181 | ATC Diagnosis Proxies | R05 | Cough and cold preparations |
| 125 | 0.00180 | ATC Diagnosis Proxies | J07 | Vaccines |
| 126 | 0.00178 | ICD-10 Diagnosis | R55 | Syncope and collapse |
| 127 | 0.00177 | ATC Diagnosis Proxies | M03 | Muscle relaxants |
| 128 | 0.00177 | ICD-10 Diagnosis | N84 | Polyp of female genital tract |
| 129 | 0.00176 | ICD-10 Diagnosis | M51 | Thoracic, thoracolum, and lumbosacral intvrt disc disorders |
| 130 | 0.00171 | ICD-10 Diagnosis | K58 | Irritable bowel syndrome |
| 131 | 0.00170 | ATC Fall Risk | C02CA | Alpha-adrenoreceptor antagonists |
| 132 | 0.00169 | ATC Fall Risk | N03AX | Other antiepileptics |
| 133 | 0.00169 | ICD-10 Diagnosis | R25 | Abnormal involuntary movements |
| 134 | 0.00160 | ICD-10 Diagnosis | H93 | Other disorders of ear, not elsewhere classified |
| 135 | 0.00158 | ICD-10 Diagnosis | I69 | Sequelae of cerebrovascular disease |
| 136 | 0.00154 | ATC Diagnosis Proxies | A02 | Drugs for acid related disorders |
| 137 | 0.00154 | ATC Diagnosis Proxies | L04 | Immunosuppressants |
| 138 | 0.00153 | ATC Fall Risk (Redemptions within 6 Months) | C09CA | Angiotensin ii receptor blockers (arbs), plain |
| 139 | 0.00152 | ATC Diagnosis Proxies | C03 | Diuretics |
| 140 | 0.00152 | ICD-10 Diagnosis | T84 | Complications of internal orthopaedic prosthetic devices, implants or grafts |
| 141 | 0.00151 | ATC Diagnosis Proxies | C08 | Calcium channel blockers |
| 142 | 0.00150 | ICD-10 Diagnosis | O72 | Postpartum hemorrhage |
| 143 | 0.00150 | ATC Diagnosis Proxies | M02 | Topical products for joint and muscular pain |
| 144 | 0.00147 | ICD-10 Diagnosis | E68 | Sequelae of hyperalimentation |
| 145 | 0.00147 | ICD-10 Diagnosis | M23 | Internal derangement of knee |
| 146 | 0.00146 | ATC Fall Risk | N05AN | Lithium |
| 147 | 0.00143 | ATC Diagnosis Proxies | A08 | Antiobesity preparations, excl. diet products |
| 148 | 0.00143 | ICD-10 Diagnosis | M17 | Osteoarthritis of knee |
| 149 | 0.00142 | ICD-10 Diagnosis | R63 | Symptoms and signs concerning food and fluid intake |
| 150 | 0.00140 | ICD-10 Risk Factors |  | Mild Liver Disease |
| 151 | 0.00139 | ATC Diagnosis Proxies | G03 | Sex hormones and modulators of the genital system |
| 152 | 0.00139 | ICD-10 Diagnosis | T92 | Sequelae of injuries of upper limb |
| 153 | 0.00139 | ICD-10 Diagnosis | L82 | Seborrheic keratosis |
| 154 | 0.00139 | ATC Diagnosis Proxies | J05 | Antivirals for systemic use |
| 155 | 0.00138 | ICD-10 Diagnosis | K22 | Other diseases of esophagus |
| 156 | 0.00138 | ATC Fall Risk | C08DA | Phenylalkylamine derivatives |
| 157 | 0.00136 | ICD-10 Diagnosis | S70 | Superficial injury of hip and thigh |
| 158 | 0.00126 | ICD-10 Diagnosis | E04 | Other nontoxic goiter |
| 159 | 0.00126 | ATC Diagnosis Proxies | D07 | Corticosteroids, dermatological preparations |
| 160 | 0.00126 | ICD-10 Diagnosis | M25 | Other joint disorder, not elsewhere classified |
| 161 | 0.00126 | ATC Diagnosis Proxies | S02 | Otologicals |
| 162 | 0.00125 | ICD-10 Diagnosis | A41 | Other sepsis |
| 163 | 0.00124 | ICD-10 Diagnosis | M54 | Dorsalgia |
| 164 | 0.00124 | ICD-10 Diagnosis | R41 | Oth symptoms and signs with cognitive functions and awareness |
| 165 | 0.00123 | ICD-10 Diagnosis | I70 | Atherosclerosis |
| 166 | 0.00122 | ATC Osteoporosis Risk | N06AX | Other antidepressants |
| 167 | 0.00121 | ICD-10 Diagnosis | R52 | Pain, unspecified |
| 168 | 0.00120 | ATC Osteoporosis Risk | B01AA | Vitamin k antagonists |
| 169 | 0.00120 | ATC Fall Risk | C07AA | Beta blocking agents, non-selective |
| 170 | 0.00120 | ICD-10 Diagnosis | C18 | Malignant neoplasm of colon |
| 171 | 0.00120 | ICD-10 Diagnosis | S40 | Superficial injury of shoulder and upper arm |
| 172 | 0.00119 | ICD-10 Diagnosis | K52 | Other and unspecifiednoninfective gastroenteritis and colitis |
| 173 | 0.00119 | ICD-10 Diagnosis | R33 | Retention of urine |
| 174 | 0.00118 | ATC Diagnosis Proxies | R06 | Antihistamines for systemic use |
| 175 | 0.00117 | ICD-10 Diagnosis | K50 | Crohn's disease [regional enteritis] |
| 176 | 0.00115 | ICD-10 Diagnosis | K56 | Paralytic ileus and intestinal obstruction without hernia |
| 177 | 0.00114 | ICD-10 Diagnosis | T81 | Complications of procedures, not elsewhere classified |
| 178 | 0.00114 | ATC Fall Risk | N05AF | Thioxanthene derivatives |
| 179 | 0.00114 | ATC Fall Risk | N03AF | Carboxamide derivatives |
| 180 | 0.00113 | ICD-10 Diagnosis | D24 | Benign neoplasm of breast |
| 181 | 0.00113 | ATC Diagnosis Proxies | A09 | Digestives, incl. enzymes |
| 182 | 0.00112 | ICD-10 Diagnosis | I10 | Essential (primary) hypertension |
| 183 | 0.00112 | ICD-10 Diagnosis | H52 | Disorders of refraction and accommodation |
| 184 | 0.00110 | ICD-10 Diagnosis | M35 | Other systemic involvement of con, not elsewhere classifiedtive tissue |
| 185 | 0.00109 | ICD-10 Diagnosis | I71 | Aortic aneurysm and dissection |
| 186 | 0.00109 | ICD-10 Diagnosis | I50 | Heart failure |
| 187 | 0.00106 | ICD-10 Diagnosis | D27 | Benign neoplasm of ovary |
| 188 | 0.00105 | ICD-10 Diagnosis | N50 | Other and unspecified disorders of male genital organs |
| 189 | 0.00104 | ICD-10 Diagnosis | H33 | Retinal detachments and breaks |
| 190 | 0.00104 | ICD-10 Diagnosis | S51 | Open wound of elbow and forearm |
| 191 | 0.00103 | ICD-10 Diagnosis | J18 | Pneumonia, unspecified organism |
| 192 | 0.00103 | ICD-10 Diagnosis | T15 | Foreign body on external eye |
| 193 | 0.00102 | ICD-10 Diagnosis | M21 | Other acquired deformities of limbs |
| 194 | 0.00100 | ICD-10 Diagnosis | F43 | Reaction to severe stress, and adjustment disorders |
| 195 | 0.00100 | ICD-10 Diagnosis | D50 | Iron deficiency anemia |
| 196 | 0.00099 | ATC Fall Risk | N05AX | Other antipsychotics |
| 197 | 0.00098 | ICD-10 Diagnosis | H50 | Other strabismus |
| 198 | 0.00098 | ATC Diagnosis Proxies | H02 | Corticosteroids for systemic use |
| 199 | 0.00098 | ICD-10 Diagnosis | K21 | Gastro-esophageal reflux disease |
| 200 | 0.00097 | ATC Diagnosis Proxies | A07 | Antidiarrheals, intestinal antiinflammatory/antiinfective agents |
| 201 | 0.00097 | ICD-10 Diagnosis | R50 | Fever of other and unknown origin |
| 202 | 0.00095 | ICD-10 Diagnosis | S61 | Open wound of wrist, hand and fingers |
| 203 | 0.00095 | ICD-10 Diagnosis | T79 | Certain early complications of trauma, not elsewhere classified |
| 204 | 0.00095 | ICD-10 Risk Factors |  | Current Smoker |
| 205 | 0.00095 | ICD-10 Diagnosis | K57 | Diverticular disease of intestine |
| 206 | 0.00094 | ATC Diagnosis Proxies | B02 | Antihemorrhagics |
| 207 | 0.00092 | ICD-10 Diagnosis | J30 | Vasomotor and allergic rhinitis |
| 208 | 0.00091 | ATC Diagnosis Proxies | A04 | Antiemetics and antinauseants |
| 209 | 0.00090 | ATC Diagnosis Proxies | P02 | Anthelmintics |
| 210 | 0.00089 | ICD-10 Diagnosis | R79 | Other abnormal findings of blood chemistry |
| 211 | 0.00088 | ICD-10 Diagnosis | B34 | Viral infection of unspecified site |
| 212 | 0.00088 | ICD-10 Diagnosis | I67 | Other cerebrovascular diseases |
| 213 | 0.00087 | ATC Diagnosis Proxies | H04 | Pancreatic hormones |
| 214 | 0.00087 | ICD-10 Diagnosis | L98 | Oth disorders of skin, subcu, not elsewhere classified |
| 215 | 0.00087 | ATC Fall Risk | C02AC | Imidazoline receptor agonists |
| 216 | 0.00083 | ICD-10 Diagnosis | I21 | Acute myocardial infarction |
| 217 | 0.00083 | ICD-10 Diagnosis | I83 | Varicose veins of lower extremities |
| 218 | 0.00082 | ICD-10 Diagnosis | G50 | Disorders of trigeminal nerve |
| 219 | 0.00081 | ICD-10 Diagnosis | K08 | Other disorders of teeth and supporting structures |
| 220 | 0.00081 | ICD-10 Diagnosis | C44 | Other and unspecified malignant neoplasm of skin |
| 221 | 0.00080 | ICD-10 Diagnosis | K63 | Other diseases of intestine |
| 222 | 0.00080 | ICD-10 Diagnosis | N30 | Cystitis |
| 223 | 0.00079 | ICD-10 Diagnosis | G56 | Mononeuropathies of upper limb |
| 224 | 0.00079 | ATC Fall Risk | M03BX | Other centrally acting agents |
| 225 | 0.00078 | ICD-10 Diagnosis | S91 | Open wound of ankle, foot and toes |
| 226 | 0.00076 | ICD-10 Diagnosis | S83 | Dislocation and sprain of joints and ligaments of knee |
| 227 | 0.00076 | ICD-10 Diagnosis | M18 | Osteoarthritis of first carpometacarpal joint |
| 228 | 0.00075 | ATC Diagnosis Proxies | C09 | Agents acting on the renin-angiotensin system |
| 229 | 0.00075 | ICD-10 Diagnosis | G45 | Transient cerebral ischemic attacks and related syndromes |
| 230 | 0.00074 | ICD-10 Diagnosis | R67 | Findings during assessment of general functional ability |
| 231 | 0.00073 | ICD-10 Risk Factors |  | Multiple Sclerosis |
| 232 | 0.00073 | ICD-10 Diagnosis | K81 | Cholecystitis |
| 233 | 0.00072 | ICD-10 Diagnosis | H81 | Disorders of vestibular function |
| 234 | 0.00072 | ICD-10 Diagnosis | R61 | Generalized hyperhidrosis |
| 235 | 0.00071 | ICD-10 Diagnosis | R39 | Oth and unspecifiedsymptoms and signs involving the GU sys |
| 236 | 0.00071 | ICD-10 Diagnosis | K62 | Other diseases of anus and rectum |
| 237 | 0.00071 | ICD-10 Diagnosis | L73 | Other follicular disorders |
| 238 | 0.00070 | ICD-10 Diagnosis | E21 | Hyperparathyroidism and other disorders of parathyroid gland |
| 239 | 0.00069 | ICD-10 Diagnosis | B37 | Candidiasis |
| 240 | 0.00069 | ICD-10 Diagnosis | M77 | Other enthesopathies |
| 241 | 0.00068 | ICD-10 Diagnosis | T26 | Burn and corrosion confined to eye and adnexa |
| 242 | 0.00068 | ICD-10 Diagnosis | K30 | Functional dyspepsia |
| 243 | 0.00066 | ATC Diagnosis Proxies | A01 | Stomatological preparations |
| 244 | 0.00066 | ICD-10 Diagnosis | H04 | Disorders of lacrimal system |
| 245 | 0.00066 | ICD-10 Diagnosis | M43 | Other deforming dorsopathies |
| 246 | 0.00065 | ATC Fall Risk (Redemptions within 6 Months) | C09AA | Ace inhibitors, plain |
| 247 | 0.00065 | ICD-10 Diagnosis | J20 | Acute bronchitis |
| 248 | 0.00062 | ATC Osteoporosis Risk | N03AF | Carboxamide derivatives |
| 249 | 0.00062 | ICD-10 Diagnosis | R31 | Hematuria |
| 250 | 0.00060 | ATC Fall Risk | N03AE | Benzodiazepine derivatives |
| 251 | 0.00060 | ICD-10 Diagnosis | E83 | Disorders of mineral metabolism |
| 252 | 0.00060 | ATC Fall Risk | N05CD | Benzodiazepine derivatives |
| 253 | 0.00059 | ICD-10 Diagnosis | I80 | Phlebitis and thrombophlebitis |
| 254 | 0.00058 | ICD-10 Diagnosis | H36 | Retinal disorders in diseases classified elsewhere |
| 255 | 0.00058 | ICD-10 Diagnosis | M22 | Disorder of patella |
| 256 | 0.00056 | ICD-10 Diagnosis | R59 | Enlarged lymph nodes |
| 257 | 0.00056 | ICD-10 Diagnosis | E16 | Other disorders of pancreatic internal secretion |
| 258 | 0.00055 | ICD-10 Diagnosis | H00 | Hordeolum and chalazion |
| 259 | 0.00054 | ICD-10 Diagnosis | G25 | Other extrapyramidal and movement disorders |
| 260 | 0.00053 | ATC Fall Risk (Redemptions within 6 Months) | C07AB | Beta blocking agents, selective |
| 261 | 0.00053 | ICD-10 Diagnosis | N20 | Calculus of kidney and ureter |
| 262 | 0.00052 | ICD-10 Diagnosis | Q61 | Cystic kidney disease |
| 263 | 0.00052 | ATC Diagnosis Proxies | A03 | Drugs for functional gastrointestinal disorders |
| 264 | 0.00051 | ATC Fall Risk (Redemptions within 6 Months) | C03AB | Thiazides and potassium in combination |
| 265 | 0.00051 | ICD-10 Diagnosis | S02 | Fracture of skull and facial bones |
| 266 | 0.00051 | ICD-10 Diagnosis | M05 | Rheumatoid arthritis with rheumatoid factor |
| 267 | 0.00050 | ICD-10 Diagnosis | I49 | Other cardiac arrhythmias |
| 268 | 0.00049 | ATC Fall Risk | C07AG | Alpha and beta blocking agents |
| 269 | 0.00049 | ICD-10 Diagnosis | D22 | Melanocytic nevi |
| 270 | 0.00049 | ICD-10 Diagnosis | B02 | Zoster [herpes zoster] |
| 271 | 0.00047 | ICD-10 Diagnosis | S46 | Injury of muscle, fascia and tendon at shldr/up arm |
| 272 | 0.00047 | ATC Diagnosis Proxies | M04 | Antigout preparations |
| 273 | 0.00047 | ATC Fall Risk | C03EA | Low-ceiling diuretics and potassium-sparing agents |
| 274 | 0.00046 | ICD-10 Diagnosis | L97 | Non-pressure chronic ulcer of lower limb, not elsewhere classified |
| 275 | 0.00045 | ICD-10 Diagnosis | I25 | Chronic ischemic heart disease |
| 276 | 0.00045 | ICD-10 Diagnosis | K11 | Diseases of salivary glands |
| 277 | 0.00045 | ICD-10 Diagnosis | S33 | Dislocation and sprain of joints and ligaments of lumbar spine and pelvis |
| 278 | 0.00043 | ICD-10 Risk Factors |  | Diabetes Mellitius |
| 279 | 0.00043 | ICD-10 Diagnosis | F17 | Nicotine dependence |
| 280 | 0.00042 | ATC Fall Risk | C03AA | Thiazides, plain |
| 281 | 0.00042 | ICD-10 Diagnosis | G43 | Migraine |
| 282 | 0.00041 | ICD-10 Diagnosis | M99 | Biomechanical lesions, not elsewhere classified |
| 283 | 0.00041 | ICD-10 Diagnosis | H54 | Blindness and low vision |
| 284 | 0.00039 | ATC Fall Risk | N05BB | Diphenylmethane derivatives |
| 285 | 0.00039 | ICD-10 Risk Factors |  | Renal Disease |
| 286 | 0.00039 | ICD-10 Risk Factors |  | Underweight |
| 287 | 0.00039 | ICD-10 Diagnosis | D25 | Leiomyoma of uterus |
| 288 | 0.00039 | ATC Fall Risk (Redemptions within 6 Months) | C09BA | Ace inhibitors and diuretics |
| 289 | 0.00039 | ICD-10 Diagnosis | I82 | Other venous embolism and thrombosis |
| 290 | 0.00036 | ICD-10 Diagnosis | N94 | Pain and other cond assoc with fem gntl org and menstrual cycle |
| 291 | 0.00036 | ICD-10 Diagnosis | E03 | Other hypothyroidism |
| 292 | 0.00035 | ICD-10 Diagnosis | M62 | Other disorders of muscle |
| 293 | 0.00035 | ICD-10 Diagnosis | T88 | Oth complications of surgical and medical care, not elsewhere classified |
| 294 | 0.00035 | ATC Diagnosis Proxies | N01 | Anesthetics |
| 295 | 0.00034 | ICD-10 Diagnosis | K26 | Duodenal ulcer |
| 296 | 0.00034 | ICD-10 Diagnosis | G31 | Oth degenerative diseases of nervous system, not elsewhere classified |
| 297 | 0.00034 | ICD-10 Diagnosis | D23 | Other benign neoplasms of skin |
| 298 | 0.00034 | ICD-10 Diagnosis | T83 | Complications of genitourinary prosthetic devices, implants or grafts |
| 299 | 0.00034 | ICD-10 Diagnosis | E11 | Type 2 diabetes mellitus |
| 300 | 0.00034 | ATC Diagnosis Proxies | C01 | Cardiac therapy |
| 301 | 0.00033 | ICD-10 Risk Factors |  | Chronic Pulmonary Disease |
| 302 | 0.00032 | ICD-10 Diagnosis | S23 | Dislocation and sprain of joints and ligaments of thorax |
| 303 | 0.00032 | ICD-10 Risk Factors |  | Primary Hyperparathyroidism |
| 304 | 0.00032 | ATC Diagnosis Proxies | G01 | Gynecological antiinfectives and antiseptics |
| 305 | 0.00031 | ATC Fall Risk (Redemptions within 6 Months) | N06AB | Selective serotonin reuptake inhibitors |
| 306 | 0.00030 | ICD-10 Diagnosis | I64 | Stroke, not specified as haemorrhage or infarction |
| 307 | 0.00030 | ICD-10 Diagnosis | T63 | Toxic effect of contact with venomous animals and plants |
| 308 | 0.00030 | ICD-10 Diagnosis | M13 | Other arthritis |
| 309 | 0.00030 | ICD-10 Diagnosis | D17 | Benign lipomatous neoplasm |
| 310 | 0.00030 | ICD-10 Diagnosis | N89 | Other noninflammatory disorders of vagina |
| 311 | 0.00029 | ICD-10 Diagnosis | K65 | Peritonitis |
| 312 | 0.00029 | ICD-10 Diagnosis | R51 | Headache |
| 313 | 0.00028 | ICD-10 Diagnosis | H90 | Conductive and sensorineural hearing loss |
| 314 | 0.00028 | ICD-10 Diagnosis | H02 | Other disorders of eyelid |
| 315 | 0.00028 | ATC Diagnosis Proxies | D05 | Antipsoriatics |
| 316 | 0.00028 | ICD-10 Diagnosis | K59 | Other functional intestinal disorders |
| 317 | 0.00028 | ICD-10 Diagnosis | M06 | Other rheumatoid arthritis |
| 318 | 0.00028 | ATC Fall Risk | N05AD | Butyrophenone derivatives |
| 319 | 0.00027 | ICD-10 Diagnosis | L29 | Pruritus |
| 320 | 0.00026 | ICD-10 Diagnosis | M48 | Other spondylopathies |
| 321 | 0.00026 | ICD-10 Diagnosis | D11 | Benign neoplasm of major salivary glands |
| 322 | 0.00026 | ICD-10 Diagnosis | R45 | Symptoms and signs involving emotional state |
| 323 | 0.00026 | ICD-10 Diagnosis | M76 | Enthesopathies, lower limb, excluding foot |
| 324 | 0.00025 | ICD-10 Diagnosis | C34 | Malignant neoplasm of bronchus and lung |
| 325 | 0.00025 | ATC Diagnosis Proxies | D10 | Anti-acne preparations |
| 326 | 0.00024 | ICD-10 Diagnosis | A04 | Other bacterial intestinal infections |
| 327 | 0.00024 | ICD-10 Diagnosis | O26 | Maternal care for other conditions predom related to pregnancy |
| 328 | 0.00024 | ICD-10 Risk Factors | hythy | Hyperthyroidism |
| 329 | 0.00024 | ICD-10 Diagnosis | T98 | Sequelae of other or unspecified effects of external causes |
| 330 | 0.00024 | ICD-10 Diagnosis | T93 | Sequalae of injuries of lower limb |
| 331 | 0.00024 | ICD-10 Diagnosis | N39 | Other disorders of urinary system |
| 332 | 0.00024 | ICD-10 Diagnosis | D14 | Benign neoplasm of middle ear and respiratory system |
| 333 | 0.00024 | ICD-10 Risk Factors | malig | NA |
| 334 | 0.00024 | ICD-10 Diagnosis | N46 | Male infertility |
| 335 | 0.00023 | ICD-10 Diagnosis | H60 | Otitis externa |
| 336 | 0.00023 | ATC Osteoporosis Risk | B01AB | Heparin group |
| 337 | 0.00022 | ICD-10 Diagnosis | M70 | Soft tissue disorders related to use, overuse and pressure |
| 338 | 0.00022 | ICD-10 Diagnosis | O70 | Perineal laceration during delivery |
| 339 | 0.00022 | ATC Fall Risk | C01BD | Antiarrhythmics, class iii |
| 340 | 0.00021 | ICD-10 Diagnosis | I47 | Paroxysmal tachycardia |
| 341 | 0.00021 | ICD-10 Diagnosis | O42 | Premature rupture of membranes |
| 342 | 0.00020 | ICD-10 Diagnosis | S50 | Superficial injury of elbow and forearm |
| 343 | 0.00019 | ICD-10 Diagnosis | J15 | Bacterial pneumonia, not elsewhere classified |
| 344 | 0.00019 | ATC Fall Risk | N05AA | Phenothiazines with aliphatic side-chain |
| 345 | 0.00019 | ICD-10 Diagnosis | M24 | Other specific joint derangements |
| 346 | 0.00019 | ICD-10 Diagnosis | H83 | Other diseases of inner ear |
| 347 | 0.00019 | ICD-10 Diagnosis | S05 | Injury of eye and orbit |
| 348 | 0.00019 | ICD-10 Diagnosis | J91 | Pleural effusion in conditions classified elsewhere |
| 349 | 0.00019 | ICD-10 Diagnosis | M19 | Other and unspecified osteoarthritis |
| 350 | 0.00019 | ICD-10 Diagnosis | N43 | Hydrocele and spermatocele |
| 351 | 0.00019 | ICD-10 Diagnosis | I34 | Nonrheumatic mitral valve disorders |
| 352 | 0.00019 | ATC Fall Risk (Redemptions within 6 Months) | C01AA | Digitalis glycosides |
| 353 | 0.00019 | ATC Fall Risk | N03AA | Barbiturates and derivatives |
| 354 | 0.00019 | ICD-10 Diagnosis | M47 | Spondylosis |
| 355 | 0.00018 | ICD-10 Diagnosis | G91 | Hydrocephalus |
| 356 | 0.00018 | ATC Fall Risk | N07CA | Antivertigo preparations |
| 357 | 0.00018 | ICD-10 Diagnosis | L40 | Psoriasis |
| 358 | 0.00018 | ICD-10 Diagnosis | I95 | Hypotension |
| 359 | 0.00017 | ICD-10 Diagnosis | T13 | Other injuries of lower limb, level unspecified |
| 360 | 0.00017 | ICD-10 Diagnosis | R91 | Abnormal findings on diagnostic imaging of lung |
| 361 | 0.00017 | ATC Fall Risk (Redemptions within 6 Months) | C08CA | Dihydropyridine derivatives |
| 362 | 0.00017 | ICD-10 Diagnosis | R15 | Fecal incontinence |
| 363 | 0.00016 | ATC Fall Risk (Redemptions within 6 Months) | N05BA | Benzodiazepine derivatives |
| 364 | 0.00016 | ICD-10 Diagnosis | S81 | Open wound of knee and lower leg |
| 365 | 0.00016 | ICD-10 Diagnosis | K64 | Hemorrhoids and perianal venous thrombosis |
| 366 | 0.00016 | ICD-10 Diagnosis | M72 | Fibroblastic disorders |
| 367 | 0.00015 | ICD-10 Diagnosis | M02 | Postinfective and reactive arthropathies |
| 368 | 0.00015 | ICD-10 Diagnosis | M15 | Polyosteoarthritis |
| 369 | 0.00015 | ICD-10 Diagnosis | S43 | Disloc and sprain of joints and ligaments of shoulder girdle |
| 370 | 0.00015 | ICD-10 Diagnosis | O20 | Hemorrhage in early pregnancy |
| 371 | 0.00014 | ICD-10 Diagnosis | N10 | Acute pyelonephritis |
| 372 | 0.00014 | ICD-10 Diagnosis | A49 | Bacterial infection of unspecified site |
| 373 | 0.00014 | ATC Fall Risk | C09DX | Angiotensin ii receptor blockers (arbs), other combinations |
| 374 | 0.00013 | ICD-10 Diagnosis | K60 | Fissure and fistula of anal and rectal regions |
| 375 | 0.00013 | ICD-10 Diagnosis | G62 | Other and unspecified polyneuropathies |
| 376 | 0.00013 | ICD-10 Diagnosis | K35 | Acute appendicitis |
| 377 | 0.00013 | ICD-10 Diagnosis | I26 | Pulmonary embolism |
| 378 | 0.00013 | ICD-10 Risk Factors | rheum | Rheumatologic disease |
| 379 | 0.00013 | ICD-10 Diagnosis | I61 | Nontraumatic intracerebral hemorrhage |
| 380 | 0.00013 | ATC Fall Risk (Redemptions within 6 Months) | N05CH | Melatonin receptor agonists |
| 381 | 0.00013 | ICD-10 Diagnosis | J37 | Chronic laryngitis and laryngotracheitis |
| 382 | 0.00013 | ICD-10 Diagnosis | C79 | Secondary malignant neoplasm of other and unspecified sites |
| 383 | 0.00012 | ICD-10 Diagnosis | T07 | Unspecified multiple injuries |
| 384 | 0.00012 | ICD-10 Risk Factors | conhf | Congestive heart failure |
| 385 | 0.00012 | ICD-10 Diagnosis | R93 | Abnormal findings on diagnostic imaging of body structures |
| 386 | 0.00012 | ICD-10 Diagnosis | T18 | Foreign body in alimentary tract |
| 387 | 0.00012 | ICD-10 Diagnosis | I42 | Cardiomyopathy |
| 388 | 0.00011 | ICD-10 Diagnosis | J45 | Asthma |
| 389 | 0.00011 | ICD-10 Diagnosis | C91 | Lymphoid leukemia |
| 390 | 0.00011 | ICD-10 Diagnosis | I35 | Nonrheumatic aortic valve disorders |
| 391 | 0.00011 | ICD-10 Diagnosis | R78 | Find of drugs and other substnc, not normally found in blood |
| 392 | 0.00011 | ICD-10 Diagnosis | K55 | Vascular disorders of intestine |
| 393 | 0.00011 | ICD-10 Diagnosis | E06 | Thyroiditis |
| 394 | 0.00010 | ICD-10 Diagnosis | G54 | Nerve root and plexus disorders |
| 395 | 0.00010 | ICD-10 Diagnosis | C54 | Malignant neoplasm of corpus uteri |
| 396 | 0.00010 | ICD-10 Risk Factors | dchro | Diabetes with chronic complications |
| 397 | 0.00010 | ATC Fall Risk (Redemptions within 6 Months) | C07AG | Alpha and beta blocking agents |
| 398 | 0.00010 | ICD-10 Diagnosis | R56 | Convulsions, not elsewhere classified |
| 399 | 0.00010 | ICD-10 Diagnosis | K90 | Intestinal malabsorption |
| 400 | 0.00010 | ICD-10 Diagnosis | F10 | Alcohol related disorders |
| 401 | 0.00009 | ICD-10 Diagnosis | H53 | Visual disturbances |
| 402 | 0.00009 | ICD-10 Diagnosis | E14 | Unspecified diabetes mellitus |
| 403 | 0.00009 | ICD-10 Diagnosis | J96 | Respiratory failure, not elsewhere classified |
| 404 | 0.00009 | ICD-10 Diagnosis | R57 | Shock, not elsewhere classified |
| 405 | 0.00009 | ICD-10 Diagnosis | L43 | Lichen planus |
| 406 | 0.00009 | ICD-10 Diagnosis | S76 | Injury of muscle, fascia and tendon at hip and thigh level |
| 407 | 0.00009 | ICD-10 Diagnosis | S73 | Dislocation and sprain of joint and ligaments of hip |
| 408 | 0.00009 | ICD-10 Diagnosis | D68 | Other coagulation defects |
| 409 | 0.00008 | ICD-10 Diagnosis | N95 | Menopausal and other perimenopausal disorders |
| 410 | 0.00008 | ATC Fall Risk (Redemptions within 6 Months) | N05AH | Diazepines, oxazepines, thiazepines and oxepines |
| 411 | 0.00008 | ICD-10 Diagnosis | N62 | Hypertrophy of breast |
| 412 | 0.00008 | ICD-10 Diagnosis | L08 | Other local infections of skin and subcutaneous tissue |
| 413 | 0.00008 | ICD-10 Diagnosis | R47 | Speech disturbances, not elsewhere classified |
| 414 | 0.00007 | ICD-10 Diagnosis | B18 | Chronic viral hepatitis |
| 415 | 0.00007 | ICD-10 Diagnosis | D09 | Carcinoma in situ of other and unspecified sites |
| 416 | 0.00007 | ATC Diagnosis Proxies | A11 | Vitamins |
| 417 | 0.00007 | ICD-10 Diagnosis | K44 | Diaphragmatic hernia |
| 418 | 0.00007 | ICD-10 Diagnosis | L89 | Pressure ulcer |
| 419 | 0.00006 | ICD-10 Diagnosis | H35 | Other retinal disorders |
| 420 | 0.00006 | ICD-10 Diagnosis | S10 | Superficial injury of , not elsewhere classifiedk |
| 421 | 0.00006 | ICD-10 Diagnosis | L57 | Skin changes due to chronic expsr to nonionizing radiation |
| 422 | 0.00006 | ICD-10 Diagnosis | F00 | Dementia in Alzheimer disease |
| 423 | 0.00006 | ATC Fall Risk (Redemptions within 6 Months) | C03CA | Sulfonamides, plain |
| 424 | 0.00006 | ICD-10 Diagnosis | E86 | Volume depletion |
| 425 | 0.00006 | ICD-10 Diagnosis | N60 | Benign mammary dysplasia |
| 426 | 0.00006 | ICD-10 Diagnosis | D75 | Other and unspecifieddiseases of blood and blood-forming organs |
| 427 | 0.00006 | ATC Fall Risk | N04AB | Ethers chemically close to antihistamines |
| 428 | 0.00005 | ICD-10 Diagnosis | S53 | Dislocation and sprain of joints and ligaments of elbow |
| 429 | 0.00005 | ICD-10 Diagnosis | R11 | Nausea and vomiting |
| 430 | 0.00005 | ICD-10 Diagnosis | E89 | Postproc endocrine and metabolic comp and disorders, not elsewhere classified |
| 431 | 0.00005 | ICD-10 Risk Factors |  | Hemiplegia or paraplegia |
| 432 | 0.00005 | ICD-10 Diagnosis | G63 | Polyneuropathy in diseases classified elsewhere |
| 433 | 0.00004 | ICD-10 Diagnosis | N99 | Postprocedural disorders of genitourinary system, not elsewhere classified |
| 434 | 0.00004 | ICD-10 Diagnosis | T51 | Toxic effect of alcohol |
| 435 | 0.00004 | ICD-10 Diagnosis | C61 | Malignant neoplasm of prostate |
| 436 | 0.00004 | ICD-10 Diagnosis | J06 | Acute upper respiratory infections of multiple and unspecified sites |
| 437 | 0.00004 | ATC Fall Risk | N04AA | Tertiary amines |
| 438 | 0.00004 | ICD-10 Diagnosis | N83 | Noninflammatory disorders of ovary, fallopian tube and broad ligament |
| 439 | 0.00003 | ICD-10 Diagnosis | H80 | Otosclerosis |
| 440 | 0.00003 | ATC Fall Risk (Redemptions within 6 Months) | M03BX | Other centrally acting agents |
| 441 | 0.00003 | ATC Diagnosis Proxies | R02 | Throat preparations |
| 442 | 0.00003 | ICD-10 Diagnosis | N17 | Acute kidney failure |
| 443 | 0.00003 | ICD-10 Diagnosis | R13 | Aphagia and dysphagia |
| 444 | 0.00003 | ICD-10 Diagnosis | N28 | Oth disorders of kidney and ureter, not elsewhere classified |
| 445 | 0.00003 | ICD-10 Diagnosis | M31 | Other , not elsewhere classifiedrotizing vasculopathies |
| 446 | 0.00003 | ATC Fall Risk (Redemptions within 6 Months) | C09DA | Angiotensin ii receptor blockers (arbs) and diuretics |
| 447 | 0.00003 | ATC Fall Risk (Redemptions within 6 Months) | N03AF | Carboxamide derivatives |
| 448 | 0.00002 | ATC Fall Risk (Redemptions within 6 Months) | N06AX | Other antidepressants |
| 449 | 0.00002 | ATC Fall Risk (Redemptions within 6 Months) | C07AA | Beta blocking agents, non-selective |
| 450 | 0.00002 | ATC Fall Risk (Redemptions within 6 Months) | C03DA | Aldosterone antagonists |
| 451 | 0.00002 | ICD-10 Diagnosis | H26 | Other cataract |
| 452 | 0.00001 | ICD-10 Diagnosis | K92 | Other diseases of digestive system |
| 453 | 0.00001 | ICD-10 Diagnosis | O82 | Encounter for cesarean delivery without indication |
| 454 | 0.00001 | ICD-10 Diagnosis | K29 | Gastritis and duodenitis |
| 455 | 0.00001 | ICD-10 Diagnosis | L02 | Cutaneous abscess, furuncle and carbuncle |
| 456 | 0.00001 | ICD-10 Diagnosis | C50 | Malignant neoplasm of breast |
| 457 | 0.00001 | ICD-10 Diagnosis | F32 | Depressive episode |
| 458 | 0.00001 | ICD-10 Diagnosis | O02 | Other abnormal products of conception |
| 459 | 0.00001 | ICD-10 Diagnosis | K42 | Umbilical hernia |
| 460 | <0.00001. | ICD-10 Diagnosis | K70 | Alcoholic liver disease |
| 461 | <0.00001. | ATC Fall Risk (Redemptions within 6 Months) | G04CA | Alpha-adrenoreceptor antagonists |
| 462 | <0.00001. | ICD-10 Diagnosis | I45 | Other conduction disorders |
| 463 | <0.00001. | ICD-10 Diagnosis | N75 | Diseases of Bartholin's gland |
| 464 | <0.00001. | ICD-10 Diagnosis | R04 | Hemorrhage from respiratory passages |
| 465 | <0.00001. | ICD-10 Diagnosis | A09 | Infectious gastroenteritis and colitis, unspecified |
| 466 | <0.00001. | ICD-10 Risk Factors |  | Moderate to severe liver disease |
| 467 | <0.00001. | ICD-10 Diagnosis | A46 | Erysipelas |
| 468 | <0.00001. | ICD-10 Diagnosis | E05 | Thyrotoxicosis [hyperthyroidism] |
| 469 | <0.00001. | ICD-10 Diagnosis | H10 | Conjunctivitis |
| 470 | <0.00001. | ICD-10 Diagnosis | I48 | Atrial fibrillation and flutter |
| 471 | <0.00001. | ICD-10 Diagnosis | N47 | Disorders of prepuce |
| 472 | <0.00001. | ATC Fall Risk | R06AA | Aminoalkyl ethers |
| 473 | <0.00001. | ATC Fall Risk (Redemptions within 6 Months) | N05CF | Benzodiazepine related drugs |
| 474 | <0.00001. | ICD-10 Risk Factors |  | Menopause |
| 475 | <0.00001. | ICD-10 Diagnosis | J90 | Pleural effusion, not elsewhere classified |
| 476 | <0.00001. | ICD-10 Diagnosis | R18 | Ascites |
| 477 | <0.00001. | ICD-10 Diagnosis | R73 | Elevated blood glucose level |
| 478 | <0.00001. | ICD-10 Diagnosis | I15 | Secondary hypertension |

**Supplementary Table 11: Age- and sex-specific cutoffs in the Testing Sample**

| Age Category - Sex | Youden’s  Cutoff | Relative Risk,  corresponding to Cutoff | Cutoff for 80% Specificity | Relative Risk,  corresponding to Cutoff |
| --- | --- | --- | --- | --- |
| 45-49-Female | 0.201 | 1.21 | 0.202 | 1.21 |
| 45-49-Male | 0.187 | 1.01 | 0.211 | 1.15 |
| 50-54-Female | 0.310 | 1.06 | 0.334 | 1.15 |
| 50-54-Male | 0.228 | 1.10 | 0.240 | 1.15 |
| 55-59-Female | 0.452 | 1.11 | 0.461 | 1.13 |
| 55-59-Male | 0.254 | 1.04 | 0.284 | 1.17 |
| 60-64-Female | 0.485 | 1.01 | 0.533 | 1.11 |
| 60-64-Male | 0.305 | 1.13 | 0.313 | 1.16 |
| 65-69-Female | 0.552 | 1.01 | 0.603 | 1.10 |
| 65-69-Male | 0.392 | 1.28 | 0.361 | 1.18 |
| 70-74-Female | 0.631 | 1.05 | 0.661 | 1.10 |
| 70-74-Male | 0.409 | 1.14 | 0.429 | 1.19 |
| 75-79-Female | 0.670 | 1.04 | 0.709 | 1.10 |
| 75-79-Male | 0.479 | 1.13 | 0.511 | 1.21 |
| 80-84-Female | 0.735 | 1.02 | 0.775 | 1.08 |
| 80-84-Male | 0.560 | 1.08 | 0.611 | 1.18 |
| 85-89-Female | 0.786 | 1.01 | 0.837 | 1.07 |
| 85-89-Male | 0.677 | 1.08 | 0.716 | 1.14 |
| 90-94-Female | 0.843 | 1.03 | 0.866 | 1.06 |
| 90-94-Male | 0.720 | 1.01 | 0.783 | 1.10 |
| 95-99-Female | 0.847 | 1.03 | 0.871 | 1.06 |
| 95-99-Male | 0.780 | 1.06 | 0.805 | 1.09 |
| 100-104-Female | 0.820 | 0.98 | 0.880 | 1.06 |
| 100-104-Male | 0.800 | 1.07 | 0.808 | 1.08 |

Sample size in individuals > than 104 years were too small to estimate age- and sex-specific cutoffs. Abbreviations: MOF – major osteoporotic fracture**.**

**Supplementary Table 12: Performance of Age- and Sex-specific Cutoffs**

| type | Acc  (%) | PPV | NPV | k | Sens  (%) | Spec  (%) | F1 | Balanced  Acc (%) | TP | FP | TN | FN |  |
| --- | --- | --- | --- | --- | --- | --- | --- | --- | --- | --- | --- | --- | --- |
| Static Youden’s Cutoff (0.49) | 71.08 | 0.021 | 0.996 | 0.024 | 69.18 | 71.10 | 0.041 | 70.14 | 3,035 | 139,709 | 343,694 | 1,352 |  |
| Age- and sex-specific Youden’s Cutoff | 73.24 | 0.017 | 0.994 | 0.016 | 51.27 | 73.44 | 0.033 | 62.35 | 2,249 | 128,409 | 354,979 | 2,138 |  |
| Age- and sex-specific Cutoff for 80% Specificity | 79.57 | 0.018 | 0.993 | 0.017 | 40.37 | 79.93 | 0.034 | 60.15 | 1,771 | 97,015 | 386,373 | 2,616 |  |

Abbreviations: Acc – Accuracy; NPV – negative predictive value; PPV – positive predictive value; Sens – sensitivity; Spec – specificity; TP – true positives; FP – false positives; FN – false negatives; TP – true positives; DXA – dual x-ray absorptiometry; Osteo – Primary osteoporosis diagnosis or medication; NA – Not applicable

**Supplementary Figures**

**Supplementary Figure S1** –ROC Curve and Accuracy by Probability Cutoff for the MOF-prediction model in all individuals > 45 years.


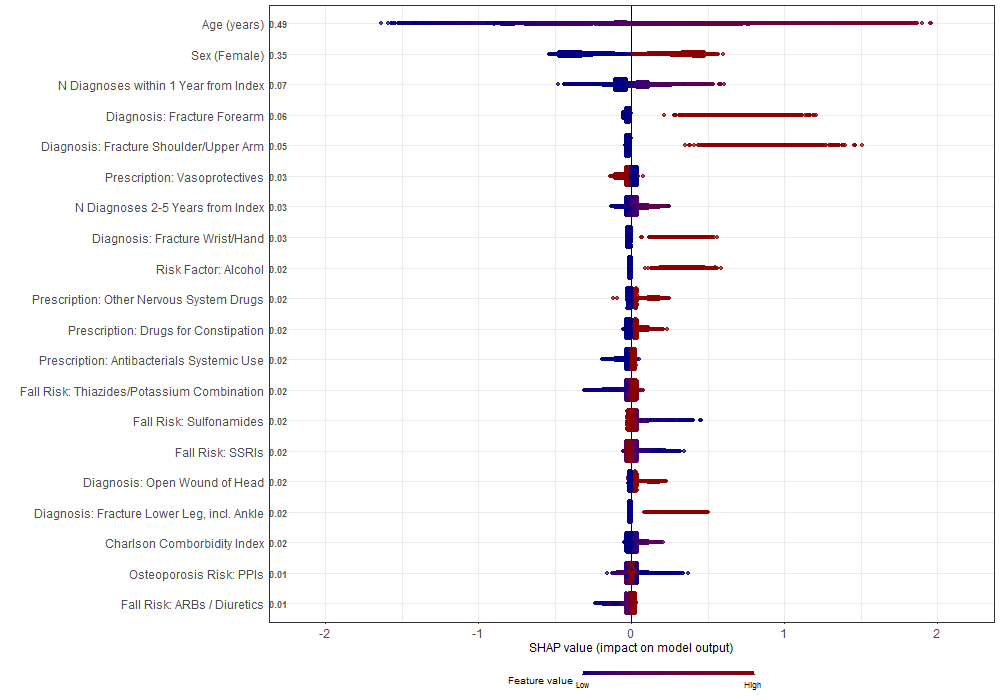


**Supplementary Figure S2** – Sina plot of SHAP values for the 20 most important features included in the non-stratified major osteoporotic fracture prediction model in the testing sample.

**Supplementary Figure S3 -** Density plots for sample distribution across predicted risk of MOF and sex in the complete sample showing the fixed non-stratified Youden’s threshold (red) and a sex-specific threshold (grey).

**Supplementary Figure S4** – Density Plot for Relative Risk of MOF sex-adjusted Risk Estimates, age-adjusted Risk Estimates, and age- and sex-adjusted Risk Estimates.

**Supplementary Figure S5** - ROC Curves for and Accuracy by Probability Cutoff for the MOF-prediction model in women (red) and men (blue) >45 years.

**Supplementary Figure S6** – Density Plot for Major Osteoporotic Fracture Probability across registered Cases in models trained separately in women (red) and men (blue).

**Supplementary Figure S7** – Barplots for mean SHAP values for the 20 most important features included in the major osteoporotic fracture model in women.

**Supplementary Figure S8** – Barplots for mean SHAP values for the 20 most important features included in the major osteoporotic fracture model in men.

**Supplementary Figure S9** – ROC Curves and Accuracy by Probability Cutoff for the Hip Fracture prediction model, as well as a Density Plot for Hip Fracture Probability across registered Case.
